# Improved tumor-only variant calling and mutation burden estimation with VarNet-T

**DOI:** 10.1038/s41467-026-71705-4

**Published:** 2026-04-09

**Authors:** Kiran Krishnamachari, Huu An Bui Nguyen, Sinem Kadioglu, Jet Ong Tze, Anders Jacobsen Skanderup

**Affiliations:** 1 Genome Institute of Singapore (GIS), Agency for Science, Technology and Research (A*STAR), Singapore, Republic of Singapore; 2 School of Computing, National University of Singapore, Singapore, Republic of Singapore

**Keywords:** Cancer genomics, Machine learning, Software, Tumour biomarkers

## Abstract

Somatic variant calling algorithms typically detect mutations in cancer genomes by comparing sequence data from a tumor sample against a matched normal sample. However, matched normal samples are often unavailable in clinical diagnostics or retrospective analyses of archival tumor samples in biobanks, compromising variant calling accuracy due to the difficulty in distinguishing somatic mutations from germline mutations or sequencing artifacts. Here, we introduce VarNet-T, an end-to-end weakly supervised deep learning framework for accurately identifying somatic variants from aligned tumor reads without a matched normal sample. VarNet-T is trained using millions of high-confidence variants and benchmarked using public datasets, demonstrating 20-33% performance improvement over existing methods. We assess the accuracy of tumor mutation burden (TMB) estimation on 1000 tumor samples spanning 10 solid cancer types. Compared to existing methods, VarNet-T demonstrates >3x higher accuracy in TMB-high status classification, suggesting significant potential to improve patient selection for immunotherapy. Overall, the improved accuracy of VarNet-T has the potential to enhance the utility of tumor-only sequencing in cancer research and clinical molecular diagnostics.

## Abstract (web summary)

Somatic variant calling in cancer normally requires matched normal samples, but the latter are often unavailable. Here, the authors develop VarNet-T, a weakly supervised deep learning framework to identify somatic variants in tumours without matched normal controls, demonstrating higher accuracy than other existing methods.

## Introduction

In 2024, an estimated 20 million new cancer cases were reported worldwide and a large number of them received next-generation sequencing (NGS) testing including targeted panels and whole exome sequencing^1^. While NGS adoption rates vary significantly across countries and cancer types, millions of patients undergo testing each year.

Somatic variant calling is the computational process used to identify somatic mutations in tumor sequencing data through various algorithms. Most existing algorithms have been designed to detect somatic mutations in matched tumor-normal sample pairs^2–4^. In research settings, a normal tissue or blood sample is typically collected in addition to the tumor sample to help identify tumor-specific mutations from those found in healthy tissue. However, matched normal samples are often unavailable in important contexts, such as NGS testing in clinical diagnostic workflows, pathology archives, cell lines, and biobanks. This negatively affects cancer treatment recommendations that often rely on accurate variant calling from tumor-only sequencing, such as immunotherapy. Tumor mutation burden (TMB), defined as the number of non-synonymous somatic mutations per megabase of the cancer genome, is a U.S. Food and Drug Administration (FDA) approved diagnostic for immune checkpoint inhibitor (ICI) therapy in multiple cancer types^5,6^. Inaccurate tumor mutation burden estimates derived from tumor-only variant calling can lead to misclassification of tumor specimens and adversely impact clinical trial results and patient outcomes^7^.

Multiple methods have been developed or adapted for tumor-only somatic variant calling. Mutect2, a commonly used variant caller, can be run in tumor-only mode. Recently, Google released Deepsomatic^8^, including models trained for tumor-only somatic variant calling. DeepSomatic uses convolutional neural networks trained on the SEQC2 benchmark dataset, derived from a cancer cell line. ClairS-TO^9^ offers deep learning models for tumor-only variant calling from long-read and short-read data, trained on a mixture of synthetic and real somatic variants derived exclusively from cancer cell lines. DeepSom^10^ is another recently published method that released multiple pre-trained and tumor-type-specific convolutional neural network models for tumor-only whole-genome somatic variant calling. DeepSom uses variant calls generated by Mutect2 (tumor-only mode) as the initial call set for prediction using its models. All these methods also leverage germline mutation databases or panels of normals for identifying non-somatic variants. Despite these advances, tumor-only somatic variant calling methods remain limited in accuracy, which compromises reliable tumor mutation burden estimation and restricts their clinical utility for immunotherapy stratification.

Here we introduce VarNet-T, a weakly supervised deep learning framework designed for somatic variant calling in tumor-only settings. In this work, we address the distinct challenges posed by the tumor-only context, where the absence of matched normal samples often leads to a significant reduction in accuracy. Existing tumor-only variant calling methods perform notably worse than their tumor-normal counterparts. We hypothesized that deep learning models trained on a large number of high-confidence somatic mutations derived from real tumor samples encoded using a representation of tumor-only sequencing reads, combined with germline variant filtering using large databases like gnomAD^11^, could enable accurate variant calling. Real tumor samples capture the biological variation and complex tumor heterogeneity, including varying tumor purity and normal cell contamination, which are characteristic of real-world biopsies and not fully captured by cell lines. This heterogeneity often also involves subclonality leading to lower variant allele frequencies (VAFs). We show that VarNet-T is able to significantly outperform existing tumor-only methods, approaching the accuracy of tumor-normal variant calling in the FDA-led SEQC2 benchmark dataset. We further demonstrate significantly improved accuracy of estimating TMB using VarNet-T, a key factor in clinical immunotherapy treatment stratification. This advancement could enhance tumor-only studies by enabling more accurate analysis of data from tumor-only biobanks, improving variant-calling accuracy and ultimately leading to better-informed treatment recommendations in clinical diagnostics.

## Results

### Overview of approach

**Fig. 1:**
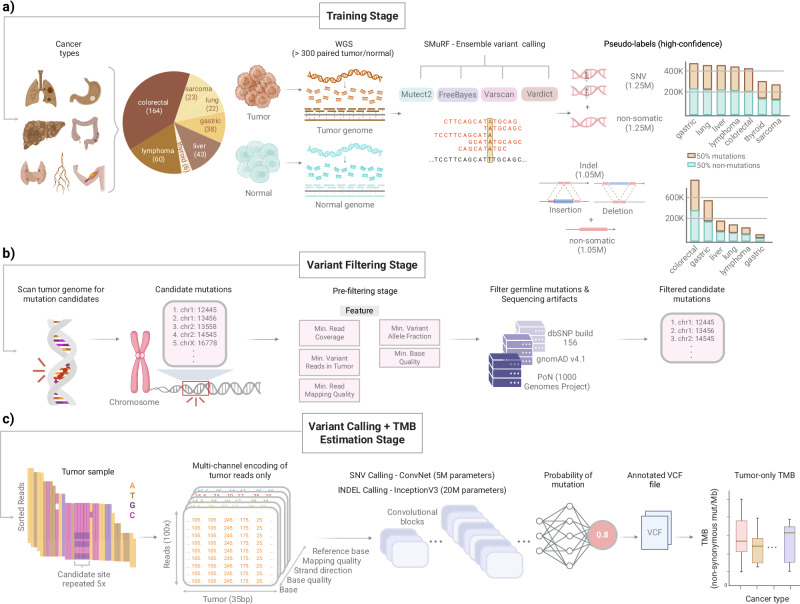
Overview of VarNet-T. **a** Training: Matched tumor/normal genomes were used to generate training data labels. Training labels were generated using high-confidence calls from 4 variant callers (via SMuRF^13^). **b** Variant Filtering: Before prediction, VarNet-T scans the tumor genome for candidate somatic mutations. Identified candidate mutations are filtered using public germline variant and panel of normal databases. Variants remaining after filtering are then classified as somatic vs non-somatic by the deep learning models. **c** Variant Calling: Each genomic position selected for training or prediction is encoded as a multi-dimensional matrix of tumor reads and associated features (e.g. base quality and mapping quality) and fed to a CNN. Annotated variant calls are then used to estimate tumor mutation burden.

In this work, we present VarNet-T, a framework for somatic variant calling in tumor-only samples without a matched normal. VarNet-T uses deep convolutional neural networks designed to process inputs lacking normal sequencing reads (see Model architecture in Methods). The training data comprised data derived from over 300 tumor-normal whole-genome sequencing pairs spanning seven distinct cancer types obtained from a previously published study^2^ (Fig. 1a). High-confidence somatic mutation calls were generated using an ensemble of existing mutation callers using matched tumor-normal samples to establish accurate training labels (see Training data in Methods). While tumor-normal pairs were used to generate training labels, the input to the deep learning model does not include normal reads. The training set included 1.25 million high-confidence somatic SNVs and 1.05 million high-confidence somatic indels, along with an equal number of non-somatic sites for each. We trained separate deep learning models for detecting somatic SNVs and indels (see Models in Methods). In the prediction stage after training, as matched germline samples are not available, VarNet-T uses public germline mutation databases to exclude germline variants. VarNet-T first scans the tumor genome to identify candidate mutations and then filters mutations found in gnomAD v4.1^11^ or dbSNP build 156^12^ (Fig. 1b). Additionally, VarNet-T uses a panel of normals to filter common sequencing artifacts (see Methods). Aligned reads overlapping each candidate mutation are encoded in an image-like multi-channel numerical representation including base, base qualities, mapping qualities, strand bias and reference base (Fig. 1c). This input representation is fed to the deep learning models for binary classification as somatic vs non-somatic.

### Benchmarking on somatic reference datasets

**Fig. 2:**
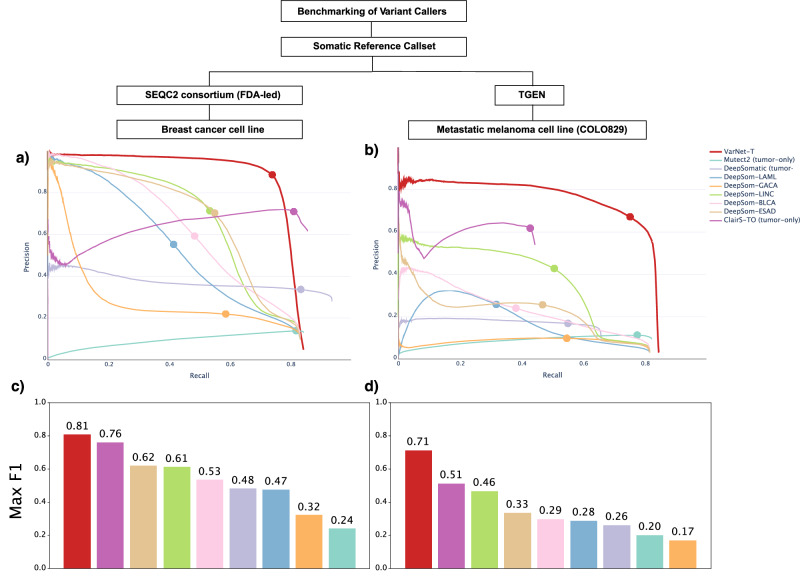
Tumor-only variant calling accuracy on benchmark tumor samples. **a**, **b** Precision-recall curves for SNV calling on the SEQC2 and COLO829 samples without using matched normal samples, respectively. VarNet-T achieves the highest AUPRC score on both samples. **c**, **d** Maximum F1 scores achieved by callers for SNV calling on the SEQC2 and COLO829 samples without using matched normal samples, respectively. Source data is provided as a Source Data file.

We benchmarked the performance of VarNet-T on independent and publicly available benchmark datasets derived from real tumor samples. Ground-truth labels in these datasets were established using both tumor and normal samples. However, we did not use the matched normal samples while running the tumor-only variant callers. We benchmarked callers on a somatic reference callset derived from a breast cancer cell line established by the FDA-led Sequencing Quality Control Phase 2 (SEQC2) consortium^14^, and COLO829^15^, a metastatic melanoma cell line with a multi-institutionally defined reference set of somatic mutations by the Translational Genomics Research Institute (TGEN). These two reference datasets were created by an ensemble approach using data from multiple sequencing and algorithmic pipelines. The SEQC2 dataset was partially validated with targeted sequencing (>2000-fold coverage) to establish high-confidence calls in the original study. On the SEQC2 dataset, VarNet-T achieved the highest Area Under the Precision-Recall Curve (AUPRC) among benchmarked callers by a significant margin (Fig. 2a, AUPRC values reported in Supplementary Table 1). VarNet-T achieved AUPRC of 0.773 on SEQC2, compared to the second highest achieved by DeepSom-ESAD (0.577). Notably, VarNet-T outperformed Deepsomatic and ClairS-TO, which are also deep learning methods but were not trained using mutations derived from real tumors. On COLO829, VarNet-T again achieved the best performing AUPRC of 0.656 (Fig. 2b), followed by DeepSom-LINC (0.318). F1 scores are reported in Fig. 2c, d. Notably, VarNet-T approaches the SNV calling performance observed in tumor-normal settings on these samples (Supplementary Fig. 1). We further analyzed the performance in different mutational contexts. The VarNet-T base model demonstrated consistent sensitivity across all single base substitution (SBS) contexts on the SEQC2 dataset. However, applying filtering using gnomAD, dbSNP, and a panel of normals caused a decrease in sensitivity, particularly for C > T transitions. This drop is likely due to the common germline and artifact nature of the C > T signature, a bias also observed in other tumor-only methods employing similar filtering (Supplementary Note 1). In insertion/deletion calling, VarNet-T again achieved the best AUPRC scores on both SEQC2 (0.527) and COLO829 (0.101). However, all callers performed less accurately on indel calling (Supplementary Fig. 2, Supplementary Table 1) as it is generally a harder problem even in the tumor-normal setting^2,16^.

### Benchmarking using whole cancer genomes from PCAWG

**Fig. 3:**
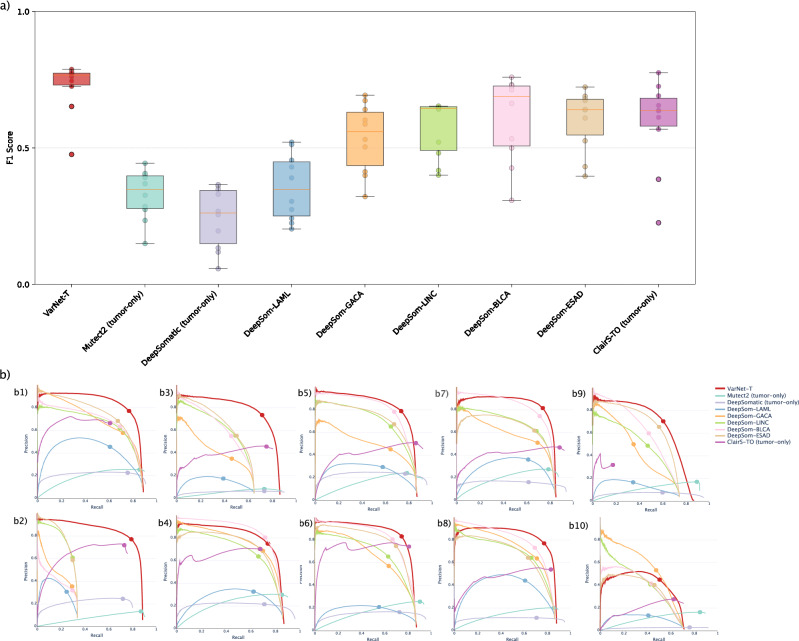
Tumor-only variant calling performance on PCAWG WGS tumor samples. **a** Box plots of the best F1 scores achieved for tumor-only SNV calling across ten PCAWG WGS samples comprising five cancer types. The box extends from the first quartile (Q1) to the third quartile (Q3) of the data, with a line at the median. The whiskers extend from the box to the farthest data point lying within 1.5× the inter-quartile range (IQR) from the box. VarNet-T achieves the highest F1 scores with least variance compared to other callers. **b** Precision-recall curves for tumor-only SNV calling on PCAWG tumor samples. Stomach Adenocarcinoma: **b1** DO38547 **b2** DO38423; Head and neck squamous cell carcinoma: **b3** DO15046 **b4** DO14566, Breast cancer: **b5** DO6144 **b6** DO1663; Bladder cancer **b7** DO804 **b8** DO522; Uterine cancer: **b9** DO41398 **b10** DO41056. Source data is provided as a Source Data file.

We further benchmarked tumor-only SNV calling accuracy using WGS samples from the PCAWG (Pan-cancer analysis of whole genomes) consortium^17^, which contains sequencing data for a large number of tumor samples across multiple cancer types. For our benchmark, we first selected five solid cancer types not used during training and then randomly selected two samples per cancer type derived from primary tumors with high somatic mutation burden (10–100k SNVs per sample). These ten samples represented five different cancer types: breast, bladder, stomach adenocarcinoma, head and neck squamous cell carcinoma, and uterine cancer. Importantly, the samples and cancer types were not included in VarNet-T’s training cohorts and hence tested VarNet-T’s generalization ability. We used expert-reviewed consensus variant calls provided by the PCAWG consortium as ground-truth sets for evaluation. VarNet-T substantially outperformed other callers (Fig. 3), achieving an average AUPRC of 0.69, performing better than Mutect2 (0.13), Deepsomatic (0.13), ClairS-TO (0.36) and all DeepSom models (DeepSom-BLCA (0.56), DeepSom-LINC (0.51), DeepSom-ESAD (0.54), DeepSom-LAML (0.20), DeepSom-GACA (0.48)). AUPRCs of each method per sample are listed in Supplementary Table 2. These results demonstrate VarNet-T’s generalization ability for tumor-only whole-genome variant calling. Notably, while DeepSom’s performance was highly variable across its models—with the worst scoring 0.20 and the best 0.56, VarNet-T’s single pan-cancer model provided better performance, eliminating the need for uncertain, cancer-specific model selection. VarNet-T also significantly outperformed other deep learning methods, Deepsomatic and ClairS-TO, on these real tumor samples. Unlike these competing methods, which were trained only on synthetic mutations or mutations derived from cancer cell lines, VarNet-T’s advantage was its training on a large number of real tumor-derived mutations using weak supervision, allowing it to capture the complex biological variation, tumor heterogeneity and normal cell contamination that cell lines lack.

### Performance at different variant allele fractions

**Fig. 4:**
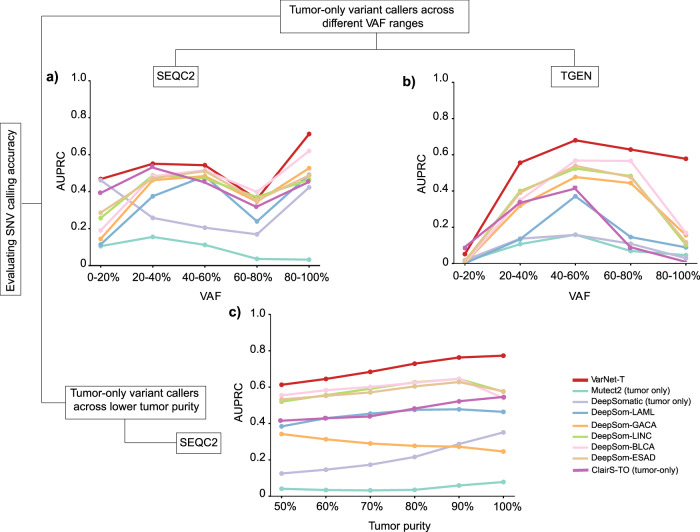
Performance at different VAF and tumor-purity levels. **a**, **b** SNV calling accuracy (AUPRC) at different VAF level ranges in the SEQC2/COLO829 benchmark samples. **c** SNV calling accuracy on SEQC2 “contaminated” with normal reads to simulate lower purity levels. Source data are provided as a Source Data file.

We evaluated the SNV calling accuracy of tumor-only variant callers at different variant allele frequency (VAF) ranges in the SEQC2 and COLO829 benchmark datasets. Both the SEQC2 and COLO829 benchmark datasets contain validated mutations at a broad range of VAFs representing both clonal and subclonal mutations. We observed VAF peaks at 100% in both ground-truth sets as these samples were derived from cell lines and hence contain no normal contamination (Supplementary Fig. 3). This may pose a challenge to tumor-only variant callers in distinguishing high-VAF somatic mutations from homozygous germline mutations, which typically also exhibit 100% VAF. On the SEQC2 dataset, VarNet-T achieves higher accuracy compared to other methods at all VAF levels except for the 60–80% VAF range (Fig. 4a). On the COLO829 dataset, VarNet-T outperforms all methods at all VAF levels (Fig. 4b). All methods achieve lower accuracy at very low VAFs (0–20%), highlighting the challenge to filter low-frequency sequencing artifacts without a matched normal sample. In summary, VarNet-T performs competitively with other tumor-only variant callers at all VAF levels contributing to its overall strong performance.

### Performance at lower tumor-purity levels

We next evaluated the accuracy of tumor-only variant callers at lower tumor-purity levels (fraction of cancer cells in tumor). The SEQC2 sample was derived from a breast cancer cell line expected to have near 100% tumor purity. Hence, we diluted this tumor sample in silico using reads from the matched normal sample to simulate decreasing levels of tumor purity (see Methods). VarNet-T outperforms other callers at all simulated tumor-purity levels (Fig. 4c, see Supplementary Fig. 4 for precision-recall curves). Most callers achieve higher accuracy with increasing tumor-purity but some callers notably experience a small dip in accuracy at 100% tumor purity possibly due to increased confusion between high-VAF somatic mutations and homozygous germline mutations. Normal cell contamination in a tumor sample is expected to reduce the variant allele fraction (VAF) of somatic mutations but not germline mutations, as the latter are present in both normal and tumor cells. Paradoxically, our analysis indicates that some normal contamination in a tumor sample may help to differentiate between high-VAF somatic mutations and germline mutations in the tumor-only setting by causing them to exhibit distinct VAF profiles.

### Estimating tumor mutation burden from tumor-only whole exome sequencing

**Fig. 5:**
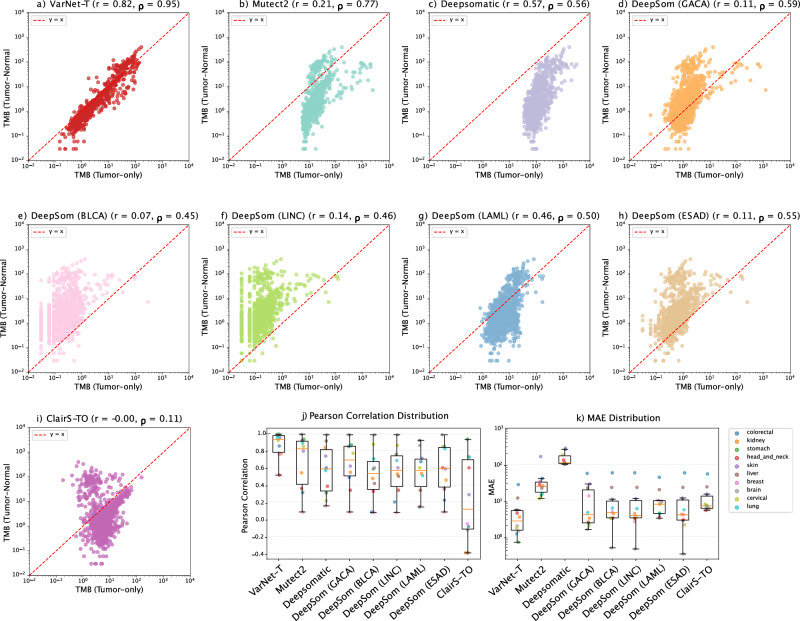
TMB estimated for 1000 TCGA WES tumor samples spanning 10 cancer types using tumor-only (x-axis) variant callers compared to a tumor-normal variant caller (y-axis). **a** VarNet-T **b** Mutect2 **c** Deepsomatic **d** DeepSom (BLCA) **e** DeepSom (LINC) **f** DeepSom (LAML) **g** DeepSom (GACA) **h** DeepSom (ESAD) **i** ClairS-TO **j** Pearson correlation and **k** Mean Absolute Error (MAE) for TMB estimates compared to tumor-normal TMB estimates. Source data are provided as a Source Data file.

Tumor mutation burden (TMB) is an FDA-approved biomarker used to guide patient stratification of immune checkpoint inhibitor (ICI) therapy across several solid tumor types^6^. A commonly used threshold is 10 mutations per megabase^6^, above which immunotherapy is often recommended due to the association between higher TMB, increased neoantigen load, and improved response to treatment. Accurate estimation of TMB is therefore critical for optimal treatment selection and improved clinical outcomes. Therefore, we estimated TMB for 1000 solid tumor-only samples from The Cancer Genome Atlas (TCGA) with available whole exome sequencing (WES) data equally distributed across ten solid cancer types (see Supplementary Table 3 for cancer-type breakdown). Since matched normal samples were also available, we established pseudo ground truth TMB estimates using tumor-normal variant calling (Supplementary Fig. 5). We then compared TMB estimates derived from tumor-only variant calls to the pseudo ground truth TMB estimates (see TMB evaluation in Methods). Existing tumor-only variant callers showed limited agreement with the tumor-normal estimates: TMB estimates using Mutect2 (tumor-only) achieved a Pearson correlation of 0.21 with tumor-normal estimates, while Deepsomatic reached 0.57. Among DeepSom’s models, DeepSom-LAML, trained on TCGA data, achieved the highest correlation of 0.46. TMB estimates using VarNet-T achieved the highest correlation (Pearson correlation of 0.82 and Spearman correlation of 0.96), demonstrating a significantly higher concordance with the tumor-normal TMB estimates (Fig. 5a–j). TMB estimates using VarNet-T also achieved the lowest Mean Absolute Error (MAE) compared to tumor-normal TMB estimates (Fig. 5k).

**Fig. 6:**
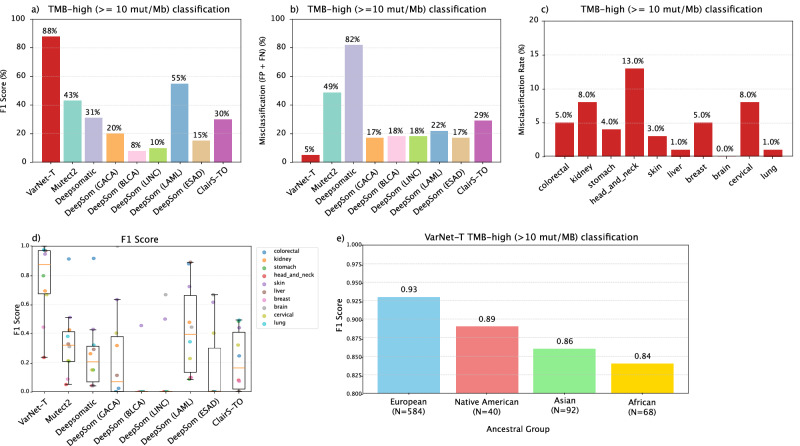
Performance metrics for TMB-high classification (≥10 mut/Mb) using tumor-only variant callers on 1000 TCGA WES tumor samples. **a** F1 accuracy in classifying TMB-high (≥10) samples, **b** Misclassification rates of methods (False Positives + False Negatives) in classifying TMB-high, **c** Misclassification rates of VarNet-T (False Positives + False Negatives) in classifying TMB-high samples per cancer type, **d** F1-scores for TMB-High classification. **e** VarNet-T’s TMB-High status (≥10 mut/MB) classification F1 accuracy by ancestral group. Source data are provided as a Source Data file.

As the key clinical decision in recommending immunotherapy is classifying a tumor as TMB-high (≥10 mut/Mb), we further assessed the accuracy of TMB estimates for identifying samples as high-TMB. VarNet-T achieved the best F1 score of 88% for this classification (Fig. 6a, d). The next best performer, DeepSom-LAML reached 55% accuracy, while Mutect2 (tumor-only) lagged behind with 43% accuracy (See Supplementary Figs. 6−15 for performance figures per cancer type). VarNet-T yielded 3x improvements over the next best method measured through misclassification rate across the entire cohort, which is the proportion of false positive and false negative predictions in TMB-high classification (Fig. 6b). Moreover, VarNet-T’s misclassification rate was 5% across all cancer types, and never exceeded 13% in any individual cancer type (Fig. 6c). This improved performance has the potential to improve treatment outcomes by correctly identifying cancers suitable for immunotherapy.

We further investigated the impact of patient genetic ancestry on the accuracy of TMB estimation by VarNet-T within this cohort. This analysis was performed on samples for which genetic ancestry annotations were available (*N* = 784)^18^. VarNet-T demonstrates robust performance in classifying TMB-high status (defined as >10 mut/MB) across diverse ancestral groups (Fig. 6e). We observed the highest estimation accuracy in samples with European ancestry, an expected finding potentially stemming from known ancestry-related reference bias in variant calling and germline mutation databases^19^. This analysis provides a strong foundation for TMB estimation and identifies a crucial area for future optimization to ensure equitable performance by mitigating systematic biases across all global populations.

### Challenges in tumor samples with low somatic mutation burden

We further evaluated the efficacy of tumor-only variant calling in tumor benchmark samples with lower mutation burden compared to SEQC2 and COLO829. The SEQC2 and COLO829 benchmark datasets contain ~39,000 and ~35,000 SNVs in their ground-truth sets, respectively. We further evaluated callers on the International Cancer Genome Consortium (ICGC)’s chronic lymphocytic leukemia (CLL) and medulloblastoma (MBL) samples^20^, and an acute myeloid leukemia (AML) sample^21^, which comprise ~1200 annotated ground-truth SNVs each and thus represent tumor-samples with an order of magnitude fewer SNVs. VarNet-T achieved the highest accuracy on these three samples. However, the accuracy of all the methods was significantly lower on these low tumor mutation burden samples (see Supplementary Fig. 16 and Supplementary Table 4). We hypothesized that tumor-only variant calling is challenging especially for samples with low tumor mutation burden, which exhibit a higher rate of false positives. To investigate this, we constructed benchmark datasets with varying tumor mutation burdens by removing known mutations in the SEQC2 sample. Our experiments revealed that variant calling performance improves with increasing tumor mutation burden due to enhanced precision (Supplementary Note 2).

VarNet-T’s lower performance in low tumor mutation burden samples is likely due to challenges in distinguishing sequencing artifacts and germline mutations without a matched normal sample. While matched normals are typically assumed to only help identify germline mutations, we hypothesized they also aid in detecting sequencing artifacts present in both tumor and normal samples due to shared sequencing processes. Removing germline variants from VarNet-T’s tumor-only calls improved accuracy, but it remained lower than VarNet’s tumor-normal performance (Supplementary Fig. 16). Strikingly, this suggests that a matched normal sample is significantly useful for filtering out sequencing artifacts. Further post-hoc analysis confirmed a significant overlap in sequencing artifacts between matched tumor and normal samples (Supplementary Note 3). This highlights that matched normals enable better identification of low-frequency and batch-specific sequencing artifacts in both sample types, improving the performance of tumor-normal variant callers.

The difficulty in distinguishing artifacts in the absence of a matched normal sample is further illustrated in Supplementary Note 4, where we show that training VarNet-T on different distributions of somatic versus non-somatic variants—even upweighting non-somatic variants to better represent a higher artifact-to-somatic ratio—resulted in poorer performance on low tumor mutation burden (TMB) samples.

### Visualizing features used by VarNet-T

**Fig. 7:**
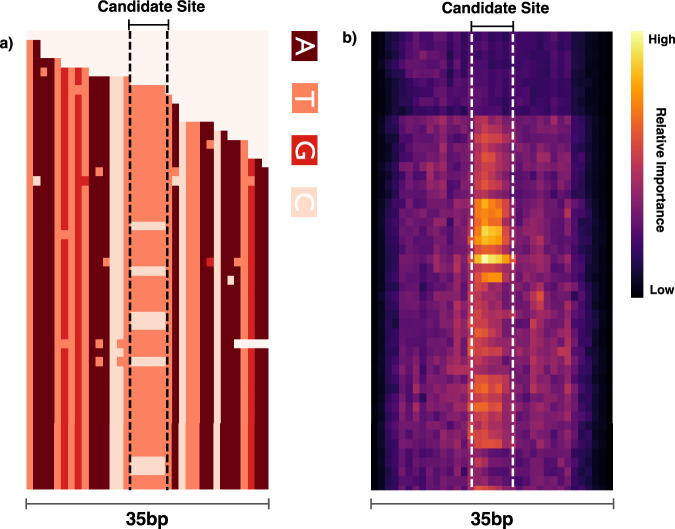
Visualization of input and pixel importance heatmap. **a** VarNet-T encoding (base channel) of a single T > C mutation in the training set. The candidate position column is repeated 5x. Variant alleles are visible in the candidate site’s column. **b** Heatmap visualization of pixel importance scores (base channel) averaged across 30 randomly selected mutations from the training set. VarNet-T pays most attention to the central candidate site column in addition to the surrounding sequence context. Pixel-wise importance scores were computed using guided backpropagation^22^.

We analyzed the features that the VarNet-T models focus on in the input. We computed the importance of input pixels using gradients of the model’s outputs with respect to its inputs, using a method called guided backpropagation^22^. We visualized the input representation used by VarNet-T (Fig. 7a) and their associated pixel importance scores (Fig. 7b). This analysis highlighted that VarNet-T is most attentive to the candidate mutation column even though it was not explicitly trained to be (Fig. 7b). Moreover, the data suggest that VarNet-T also pays attention to input pixels surrounding the candidate mutation site, which suggests that the model evaluates the sequence context of candidate mutations. It is noteworthy that VarNet-T was trained from scratch without including any specialized knowledge of mutations and yet has managed to learn important features such as variant alleles and other relevant sequence context features to solve the task.

## Discussion

Identification of somatic variants in tumor-only mode is common in the setting of clinical diagnostics and analysis of archival tumors and cell lines. We have described a method for tumor-only somatic variant calling that can substantially outperform existing methods. VarNet-T uses an end-to-end deep learning approach trained on millions of high-confidence somatic mutations from multiple cancer types. Importantly, VarNet-T was trained on real tumor-derived mutations that capture the complex biological variation, tumor heterogeneity and sub-clonality observed in real-world biopsies. Our approach also uses public germline databases and a panel of normal samples to filter out germline variants as well as common sequencing artifacts, respectively. Notably, adding germline mutations to the training set to help the model distinguish them from somatic mutations did not improve overall performance. This suggests that our filtering process using gnomAD and dbSNP is effective at removing germline variants.

We benchmarked Mutect2, DeepSom, DeepSomatic and ClairS-TO models for tumor-only variant calling. Notably, DeepSomatic, DeepSom and ClairS-TO are also deep learning-based approaches, but did not achieve the accuracy of VarNet-T in our benchmarks. VarNet-T significantly outperformed DeepSom-LAML on the acute myeloid leukemia (AML) benchmark sample, despite VarNet-T’s training data not including AML samples (unlike DeepSom-LAML, which was trained on AML). This reflects the generalization ability of VarNet-T. Notably, VarNet-T approaches the performance of tumor-normal variant calling in multiple benchmark samples (Supplementary Fig. 1).

We also examined mutational context bias in variant calling. While the VarNet-T base deep learning model maintained consistent sensitivity across all single base substitution (SBS) contexts, applying germline (gnomAD/dbSNP) and Panel-of-Normals (PoN) filters notably reduced sensitivity for C > T transitions (Supplementary Note 1). This bias stems from the high prevalence of C > T variants in both germline databases and sequencing artifacts. This highlights the trade-off in using public databases for germline exclusion.

Additionally, we examined the effect of tumor mutation burden on tumor-only variant calling, demonstrating that performance improves as mutation burden increases primarily due to a reduction in false positive calls (Supplementary Note 2).

We further analyzed the utility of a matched normal sample for filtering sequencing artifacts and germline variants, particularly in low mutation burden tumor samples where performance is still lacking. Our analysis revealed that matched normal and tumor sequencing data share a substantial number of sequencing artifacts. This overlap benefits tumor-normal variant callers and partly accounts for their superior performance compared to tumor-only callers (Supplementary Note 3). Previous studies have also explored the use of unmatched normal controls—such as panels of normals—to filter out recurrent sequencing artifacts when a matched normal sample is unavailable^23,24^. However, these studies found that matching the accuracy of a matched normal generally requires incorporating multiple unmatched normal samples. The improved performance of VarNet-T suggests that it is possible to learn features associated with many types of sequencing artifacts without relying on a matched normal control to filter them.

We further assessed the accuracy of TMB estimates using a large cohort of 1000 whole-exome sequencing samples. Existing variant callers exhibited varying degrees of correlation with the pseudo ground truth established via tumor-normal variant calling. VarNet-T demonstrated the highest correlation (Pearson r = 0.82, Spearman *ρ* = 0.96) with the ground truth. In addition, VarNet-T had >3x lower misclassification rate in identifying tumors with high TMB, based on the clinically recommended threshold of 10 non-synonymous mutations per megabase. In the clinical diagnostic setting, accurate TMB classification is critical as misclassification can lead to suboptimal treatment decisions and negatively impact patient outcomes.

In conclusion, our results improve upon existing methods for genome and exome level mutation calling from tumor-only samples, which could play a significant role in emerging clinical applications based on genome-derived biomarkers, such as TMB for immunotherapy and DNA mismatch repair (MMR) deficiency for PARP inhibitor therapies.

## Methods

### Implementation of tumor-only variant callers

We ran Mutect2 in tumor-only mode with recommended settings including a germline mutations database (gnomAD v4.1) and a panel of normals file (https://console.cloud.google.com/storage/browser/_details/gatk-best-practices/somatic-hg38/1000g_pon.hg38.vcf.gz). DeepSom (https://github.com/heiniglab/DeepSom) was run on top of Mutect2’s predictions in tumor-only mode, as recommended. DeepSom was also run with the gnomAD database and panel of normals. DeepSom uses the gnomAD database to annotate flanking germline mutations. We ran DeepSom using the five pre-trained models it includes: TCGA-LAML (acute myeloid leukemia), BLCA-US (bladder urothelial cancer), ESAD-UK (esophageal adenocarcinoma), LINC-JP (liver cancer) and GACA-CN (gastric cancer). Deepsomatic (v1.7.0) was run using the provided docker container (https://github.com/google/deepsomatic). ClairS-TO (Illumina-SSRS model, v0.4.0) was run using the provided docker image (https://github.com/HKU-BAL/ClairS-TO).

### Germline variant calling

We performed germline variant calling using DeepVariant^25^ (v1.6.1) in our analysis of shared sequencing artifacts between matched tumor and normal samples (Supplementary Note 3).

### Training data

VarNet-T was trained on data obtained from a previous study^2^. A total of 356 matched tumor and normal whole-genome sequencing samples were utilized. The specific distribution of these 356 pairs included 164 colorectal, 60 lymphoma, 23 sarcoma, 38 gastric, 22 lung, 6 thyroid and 43 liver samples. All samples were obtained with written informed consent from each patient in the original study^2^. Research protocols and sample analysis were strictly governed and approved by relevant Institutional Review Boards in the original study. Original samples were sequenced via Illumina HiSeq (50–150× depth). Reads were aligned to GRCh37 using BWA-MEM followed by marking and removal of duplicate reads. GATK3 with local realignment around indels was used for post-processing. To establish ground truth, pseudo-labels were generated using SMuRF, an ensemble somatic mutation caller that utilizes a random forest classifier informed by predictions from four individual callers (MuTect2, Freebayes somatic, VarDict, and VarScan). A total of 2.5 million SNV and 2.1 million indel datapoints were generated, with both training sets being class-balanced by designating SMuRF calls as mutated sites and specific sites missed by SMuRF but called by at least one other caller as non-mutated, intentionally creating a more challenging and discriminative learning task.

### Input representation

For each candidate mutation, VarNet-T encodes reads overlapping the genomic site from the tumor sample. Features including base, mapping quality, base quality, strand bias and reference base are encoded in separate channels of the input. Each feature is encoded using a distinct floating-point value. Deletions and insertions are also encoded in the base channel. For each candidate mutation site, input tensors of shape (100,35,5) and (140,75,5) are generated for SNVs and indels, respectively. Up to 100/140 reads can be encoded in the input to SNV/indel models, respectively. If the number of overlapping reads exceeds these limits, reads are randomly sub-sampled.

### Model architecture

VarNet-T uses convolutional neural network (CNN) architectures for both SNV and indel calling. After experimentation with multiple architecture designs, we designed a convolutional neural network (ConvNet) for SNV calling, while the InceptionV3 architecture was used for indel calling. The SNV calling model is composed as a convolutional neural network with ten convolutional blocks each containing convolution, ReLu activation and Batch Normalization^26^ layers. Two average-pooling layers are used to downsample information between blocks. Convolutional layers are followed by three densely-connected (comprising 256, 128 and 64 units) layers that are followed by a sigmoid output layer that computes the probability of mutation. There are ~3.5 million trainable parameters in the SNV model. For indel calling, a larger model, Inceptionv3^27^, was used. Both models were trained with the Adam^28^ optimizer with an initial learning rate of 1e−4 and a mini-batch size of 32. Tensorflow^29^ was used to train models on a Nvidia Titan-X GPU.

### Genome pre-filtering

VarNet-T takes as input a binary alignment map (BAM) files of a tumor-only sample. As most sites in tumor genome are not mutated and contain few or no variant-supporting reads, VarNet-T pre-filters genomic sites that have a very low likelihood of carrying a mutation. This is done to avoid the computational expense of running inference using the deep learning models on all sites in the genome. VarNet-T uses simple heuristic filters to create a list of candidate mutation sites (see Supplementary Table 5).

### Sample processing and in silico dilutions

All sequencing reads in the benchmark datasets were aligned to the GRCh38 reference genome and aligned using BWA-MEM as part of the bcbio-nextgen pipeline. For benchmarking callers on the SEQC2 reference sample^14^, we used the Illumina HiSeq sample (WGS_IL_T_1; https://sites.google.com/view/seqc2/home/sequencing) and restricted our evaluation to the high-confidence regions established by the consortium. The SEQC2 tumor sample was also mixed with its matched normal sample in various proportions using SAMtools^30^ to simulate lower tumor-purity levels. ICGC MBL, ICGC CLL and AML samples were downsampled from their original high read depths (~300×) to commonly used whole-genome sequencing read depths (~100×).

### PCAWG benchmark samples

For independent validation, we first selected five solid cancer types (Breast, Bladder, Stomach Adenocarcinoma, Head and Neck SCC, and Uterine) that were not included during training. We then randomly selected two samples per cancer type derived from primary solid tumors. This selection was specifically constrained to samples exhibiting a high, yet moderate, mutation burden (10k-100k SNVs), and all samples had a mean read coverage of 100× or less. The specific PCAWG sample IDs utilized are DO6144, DO1663 (Breast); DO522, DO804 (Bladder); DO38547, DO38423 (Stomach Adenocarcinoma); DO14566, DO15046 (Head and Neck SCC); and DO41398, DO41056 (Uterine).

### Filtering germline mutations and artifacts using public databases

VarNet-T uses public germline mutation databases to exclude germline variants from predictions. During pre-filtering, we filter any candidate mutation also found in gnomAD v4.1^11^ or any candidate mutation that is marked as a COMMON germline mutation in dbSNP build 156^12^. We filter mutations in gnomAD by both position and allele. In dbSNP, we filter by position only as we restrict dbSNP to commonly mutated sites (a COMMON SNP is one that has at least one 1000Genomes population with a minor allele of frequency ≥1% and for which 2 or more founders contribute to that minor allele frequency). We further filter artifacts using the 1000 Genomes Panel of Normals (https://console.cloud.google.com/storage/browser/_details/gatk-best-practices/somatic-hg38/1000g_pon.hg38.vcf.gz).

### Performance metrics

We have reported precision-recall curves for all methods, where *precision* is to the percentage of predicted mutations that are correct, and *recall* refers to the percentage of true mutations that were correctly identified. We varied the scores produced by each caller (VarNet’s Score, Mutect2’s TLOD, DeepSomatic’s QUAL, DeepSom’s cnn_score, ClairS-TO’s QUAL) to create precision-recall curves. We also reported F1 scores, which is the harmonic mean of precision and recall. Best F1 scores are calculated by varying the score threshold for each caller to find the optimal threshold.

### TCGA sample selection for TMB estimation

We first selected 10 solid cancer types available in TCGA. We only chose solid cancers as the TMB is an FDA-approved companion diagnostic only for solid cancers. We then randomly selected 100 WES tumor samples with matched normal samples per cancer type. To ensure that the selected samples had sufficient quality control, we only chose samples that were also part of the TCGA MC3 working group study^31^ (see Supplementary Data 1 for TCGA codes of selected tumor samples). In total, we selected 1000 solid cancers spanning 10 cancer types (Supplementary Table 3). Samples used in the training cohort were excluded from this analysis. A comprehensive list of the TCGA sample IDs utilized for training and testing can be found in the supplementary file (Supplementary Data 1). Genetic ancestry information of TCGA samples were derived from TCGA^18^ where they inferred genetic ancestry using exome sequencing or SNP array data.

### TMB calculation and evaluation

TMB was calculated as the number of non-synonymous somatic mutations per megabase of exonic regions. We used snpEff^32^ to annotate variants and identify non-synonymous mutations. The pseudo ground truth was derived from variant calls generated by VarNet^2^ on tumor-normal pairs, as VarNet is an accurate variant caller in the tumor-normal setting. To further refine the ground truth and exclude potential germline variants, we applied additional filtering using population databases (gnomAD and dbSNP). For all variant callers, TMB was computed using only variants marked as PASS. Since DeepSom does not implement default filtering or define PASS calls, and instead outputs a cnn_score for each candidate variant, we defined a threshold for the cnn_score to determine PASS calls by averaging the optimal thresholds that achieved the highest F1 scores on independent benchmark samples (SEQC2 and TGEN-COLO829).

### Reporting summary

Further information on research design is available in the Nature Portfolio Reporting Summary linked to this article.

## Supplementary information

Supplementary Information

Peer Review File

Description of Additional Supplementary Files

Supplementary Data 1

Reporting Summary

## Source data

Source data

## Data Availability

VarNet-T is publicly available at https://github.com/skandlab/VarNet under PolyForm Noncommercial License 1.0.0. To run VarNet-T, please follow the instructions to run VarNet without a matched normal sample in tumor-only mode.

## Data availability

Sequence data for all benchmark samples are from previously published studies. The MBL data are available in the European Genome-Phenome Archive (EGA) under accession code EGAD00001001859, the CLL data are available in EGA under accession code EGAD00001001858, the COLO829 data are available in EGA under accession code EGAD00001002142, the SEQC2 data are available in the Sequence Read Archive (SRA) under accession codes SRX4728512 and SRX4728509. The AML dataset was downloaded from dbGAP with accession ID phs000159 [https://www.ncbi.nlm.nih.gov/projects/gap/cgi-bin/study.cgi?study_id=phs000159]. We downloaded the sample pair with the run IDs: SRR2470200 (tumor) and SRR2177258 (normal). The 1000 TCGA WES tumor samples used for evaluating tumor mutation burden estimation are listed in supplemental file (Supplementary Data 1) and can be downloaded from GDC. Sequence data for the ten PCAWG samples used for independent validation are also available from GDC using the following uuids: 9f9bd198-13e5-4792-8d56-0937a2d79f63, a1560b03-4ef9-4fad-8d0c-3b914a0eb743, f9cb2c1c-076c-4be0-ab22-9f99e012af09, 20b79adc-1ad3-46b9-a340-7848b6338a7c, 94f20367-f7ca-428b-9e51-64335605f178, 3a66bbe7-261e-4b6c-930c-599063b90a42, 9b45b170-91c0-4054-a80c-d1ad49eecf0b, 38fd92ce-69f9-448f-8b14-8768c4bd61be, ee0644ea-bae2-43df-aa16-a13a67477afc, 1f595ce6-f7b2-46c3-af0a-d59462d5d0eb. Sequence data for the gastric cancer training cohort are from a previously published study and available from EGA under accession code EGAD00001000782, the liver cancer training cohort are also from a previously published study and available upon request through Genomic Data Commons (GDC) [https://portal.gdc.cancer.gov/projects/TCGA-LIHC]. Sequence data for the remaining training cohorts, i.e., sarcoma, lymphoma, colorectal, thyroid and lung, can be obtained from the original study^2^. Source code is described in the Code Availability section. Source data for figures and tables are provided with this paper. Source data are provided with this paper.
