# Supplementary material for: Improved tumor-only variant calling and mutation burden estimation with VarNet-T
Source: Nat Commun. 2026 Apr 9;17:5019. doi: 10.1038/s41467-026-71705-4 (PMC13243554; doi:10.1038/s41467-026-71705-4)
Supplement: Supplementary file 1 — Supplementary Information [file 41467_2026_71705_MOESM1_ESM.pdf]

Improved tumor-only variant calling and mutation burden estimation with VarNet-T

Supplementary Information

Supplementary Figure 1	1
Supplementary Figure 2	2
Supplementary Figure 3	3
Supplementary Figure 4	4
Supplementary Figure 5	5
Supplementary Figure 6	6
Supplementary Figure 7	7
Supplementary Figure 8	8
Supplementary Figure 9	9
Supplementary Figure 10	10
Supplementary Figure 11	11
Supplementary Figure 12	12
Supplementary Figure 13	13
Supplementary Figure 14	14
Supplementary Figure 15	15
Supplementary Figure 16	16
Supplementary Table 1	17
Supplementary Table 2	18
Supplementary Table 3	19
Supplementary Table 4	20
Supplementary Table 5	21
Supplementary Note 1	22
Supplementary Note 2	24
Supplementary Note 3	25
Supplementary Note 4	27

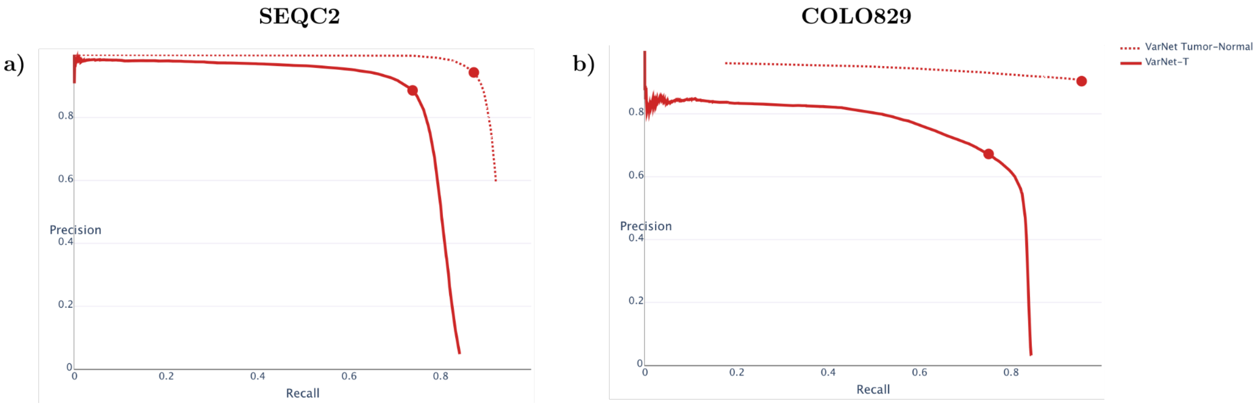

<u>Supplementary Fig. 1:</u> Performance of VarNet-T (tumor-only) compared to VarNet (tumor-normal). PR curves for SNV calling on **a** SEQC2 **b** COLO829 samples.

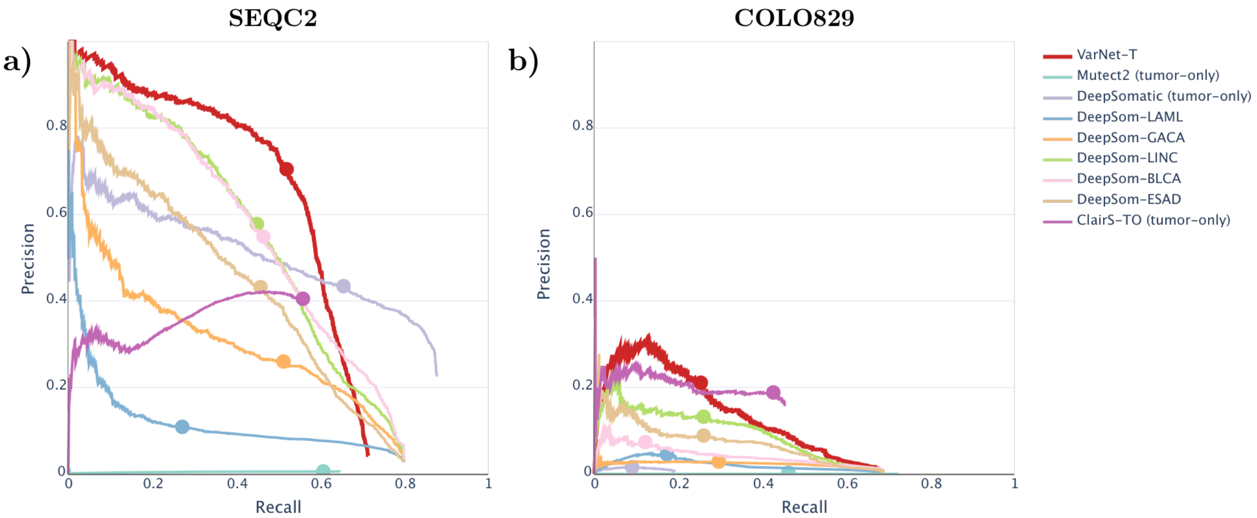

<u>Supplementary Fig. 2:</u> Precision-recall curves for indel calling on a) SEQC2 and b) COLO829 benchmark tumor samples without using matched normal samples. VarNet-T achieves the highest AUPRC score on both samples.

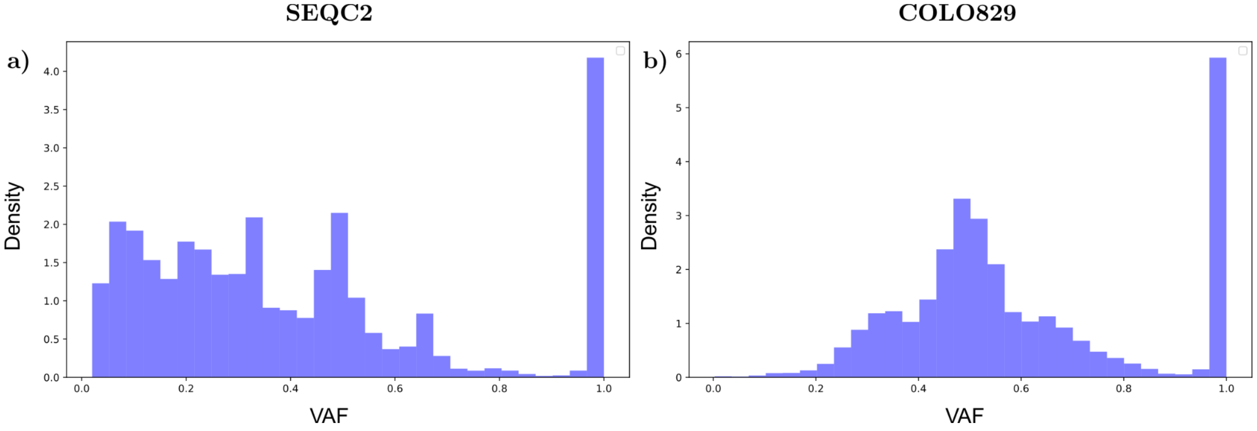

<u>Supplementary Fig. 3:</u> Variant Allele Frequency (VAF) distribution of somatic mutations (SNVs) in benchmark samples. a SEQC2 **b** COLO829.

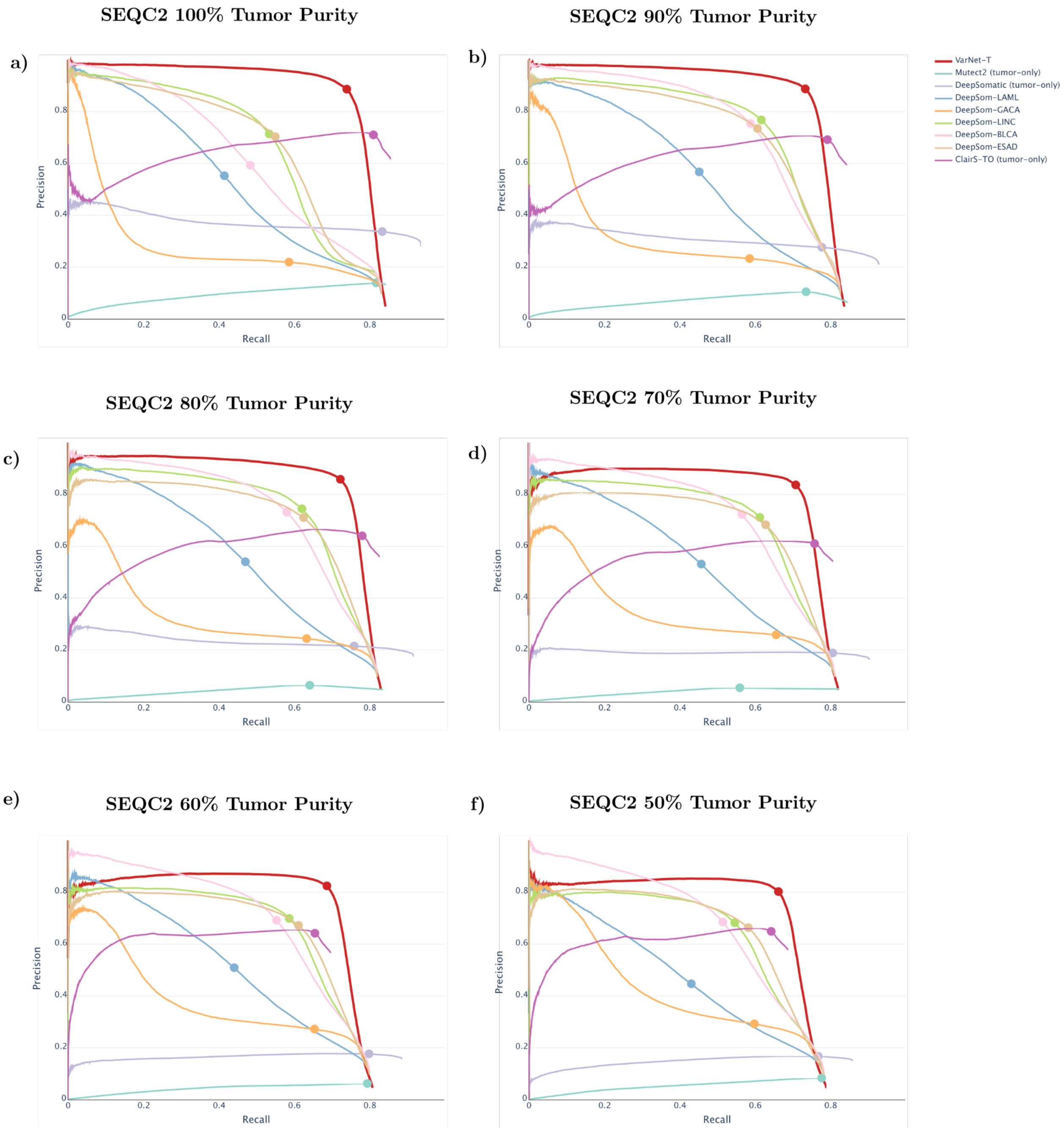

<u>Supplementary Fig. 4:</u> Performance at different tumor purity levels. PR curves for SNV calling on SEQC2 at variable tumor purity levels. Purity levels were created in silico by mixing reads from the matched normal sample. **a** 100% tumor purity. **b** 90% tumor purity. **c** 80% tumor purity. **d** 70% tumor purity. **e** 60% tumor purity. **f** 50% tumor purity.

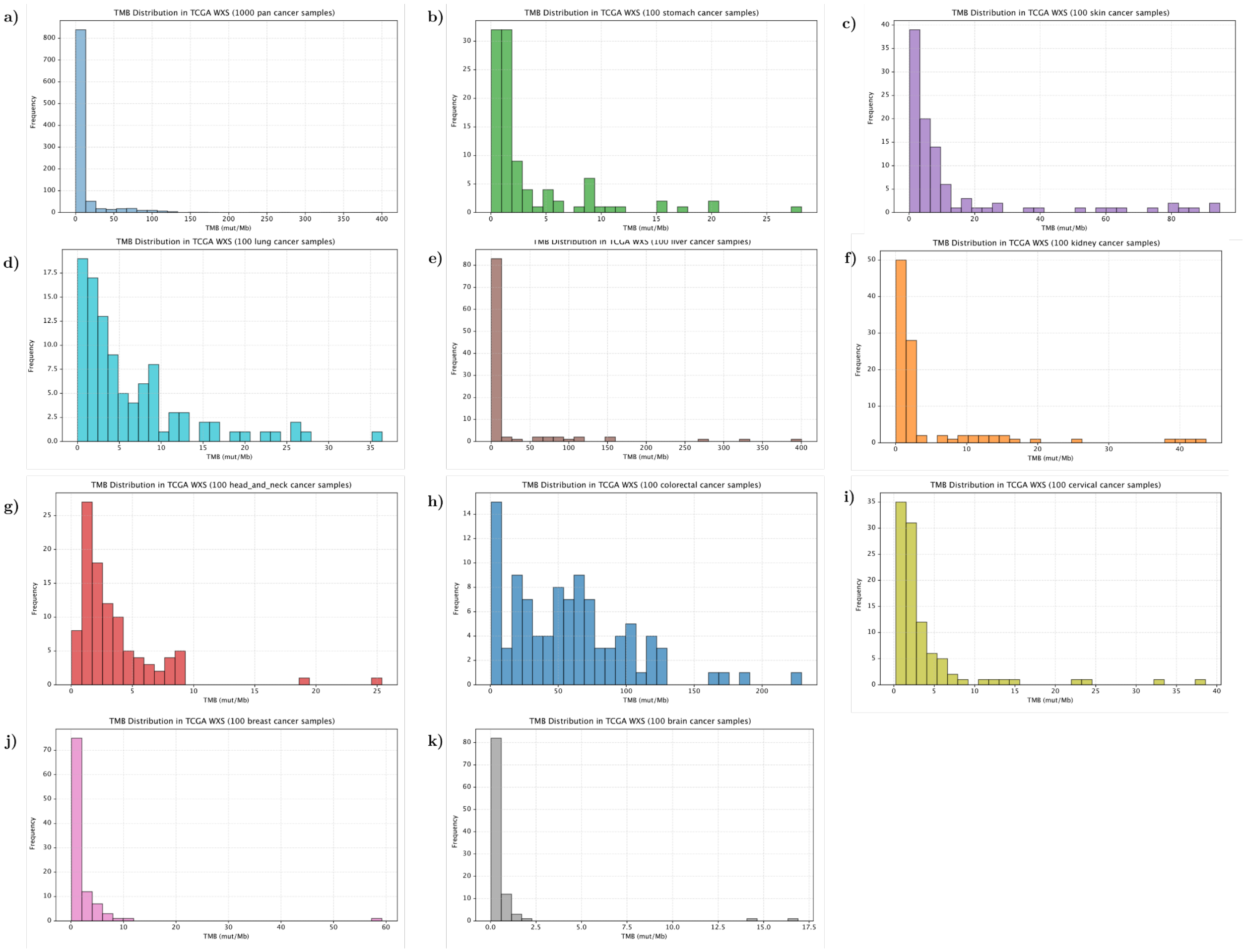

<u>Supplementary Fig. 5:</u> Tumor mutation burden (TMB) distribution in TCGA WES samples estimated using tumor-normal variant calling. a All 10 cancer types combined (100 samples per cancer type) **b** Stomach **c** Skin **d** Lung **e** Liver **f** Kidney **g** Head and Neck **h** Colorectal **i** Cervical **j** Breast **k** Brain.

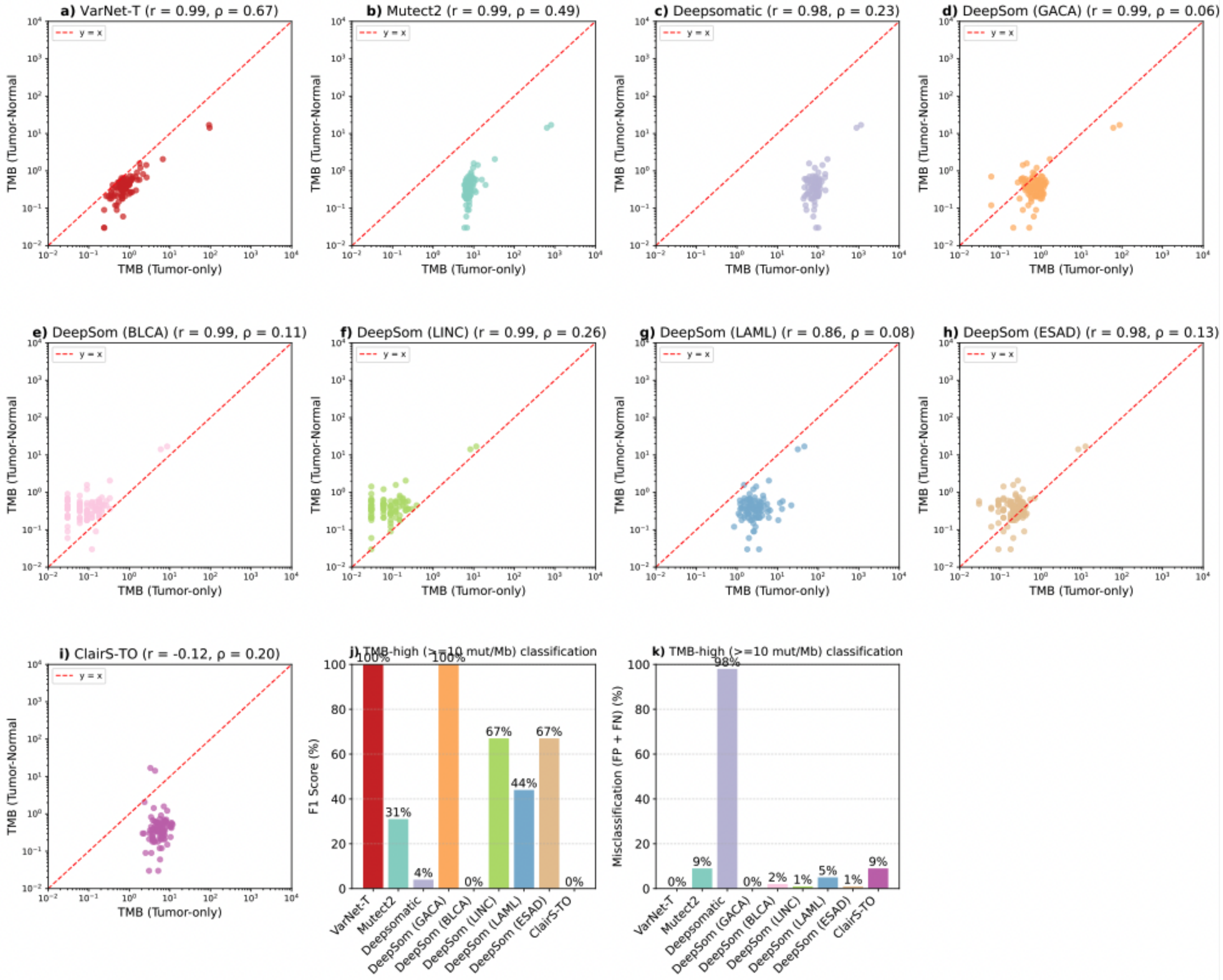

<u>Supplementary Fig. 6:</u> TMB estimated for 100 TCGA WES brain tumor samples using tumor-only (x-axis) variant callers compared to tumor-normal variant calls (y-axis). a VarNet-T **b** Mutect2 **c** Deepsomatic **d** DeepSom (BLCA) **e** DeepSom (LINC) **f** DeepSom (LAML) **g** DeepSom (GACA) **h** DeepSom (ESAD) **i** ClairS-TO **j** F1 accuracy in classifying TMB-high (≥10) samples **k** Misclassification rates (False Positives + False Negatives) in classifying TMB-high (≥10) samples. Source data are provided as a Source Data file.

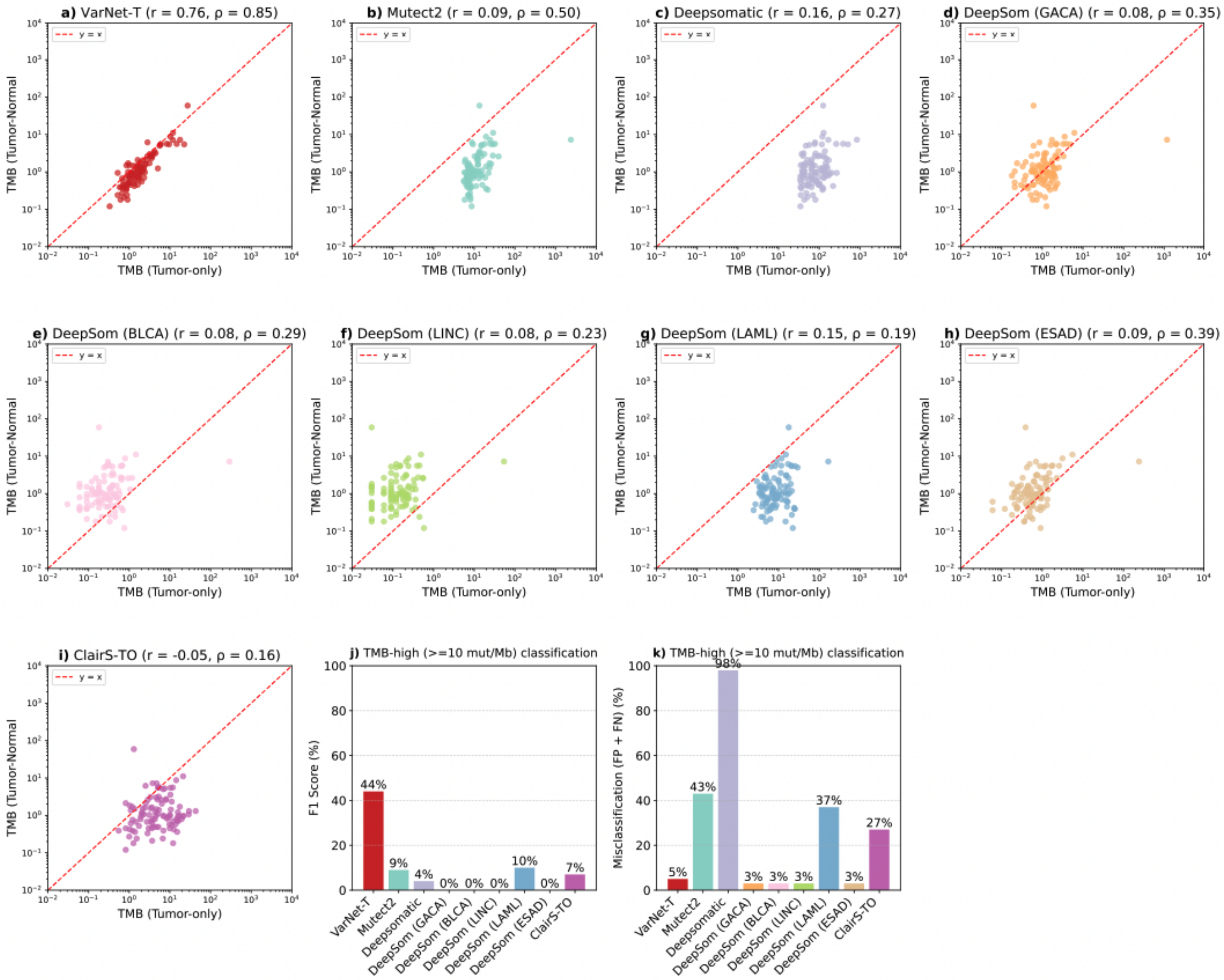

<u>Supplementary Fig. 7:</u> TMB estimated for 100 TCGA WES breast tumor samples using tumor-only (x-axis) variant callers compared to tumor-normal variant calls (y-axis). a VarNet-T **b** Mutect2 **c** Deepsomatic **d** DeepSom (BLCA) **e** DeepSom (LINC) **f** DeepSom (LAML) **g** DeepSom (GACA) **h** DeepSom (ESAD) **i** ClairS-TO **j** F1 accuracy in classifying TMB-high (≥10) samples **k** Misclassification rates (False Positives + False Negatives) in classifying TMB-high (≥10) samples. Source data are provided as a Source Data file.

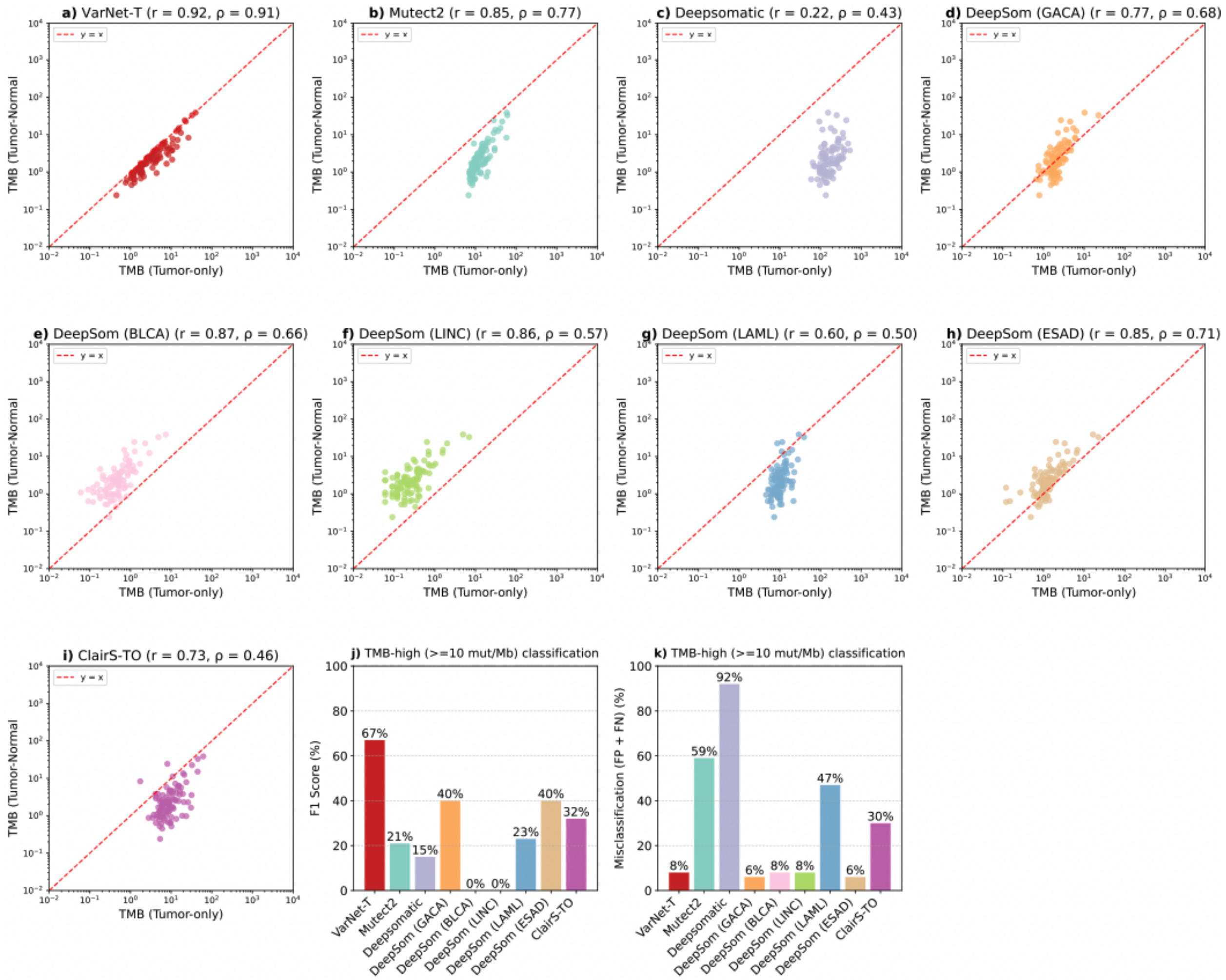

<u>Supplementary Fig. 8:</u> TMB estimated for 100 TCGA WES cervical tumor samples using tumor-only (x-axis) variant callers compared to tumor-normal variant calls (y-axis). a VarNet-T **b** Mutect2 **c** Deepsomatic **d** DeepSom (BLCA) **e** DeepSom (LINC) **f** DeepSom (LAML) **g** DeepSom (GACA) **h** DeepSom (ESAD) **i** ClairS-TO **j** F1 accuracy in classifying TMB-high (≥10) samples **k** Misclassification rates (False Positives + False Negatives) in classifying TMB-high (≥10) samples. Source data are provided as a Source Data file.

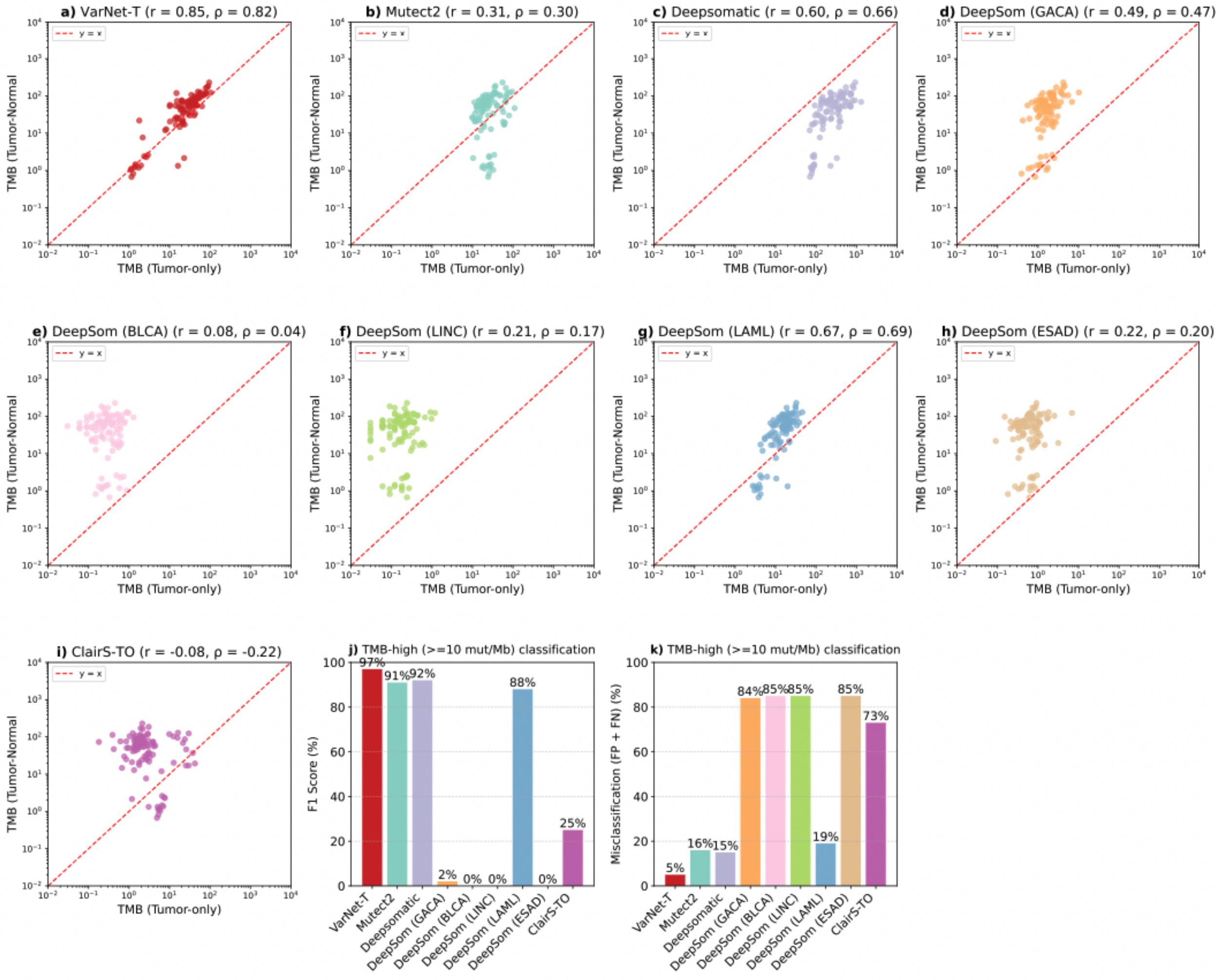

<u>Supplementary Fig. 9:</u> TMB estimated for 100 TCGA WES colorectal tumor samples using tumor-only (x-axis) variant callers compared to tumor-normal variant calls (y-axis). a VarNet-T **b** Mutect2 **c** Deepsomatic **d** DeepSom (BLCA) **e** DeepSom (LINC) **f** DeepSom (LAML) **g** DeepSom (GACA) **h** DeepSom (ESAD) **i** ClairS-TO **j** F1 accuracy in classifying TMB-high (≥10) samples **k** Misclassification rates (False Positives + False Negatives) in classifying TMB-high (≥10) samples. Source data are provided as a Source Data file.

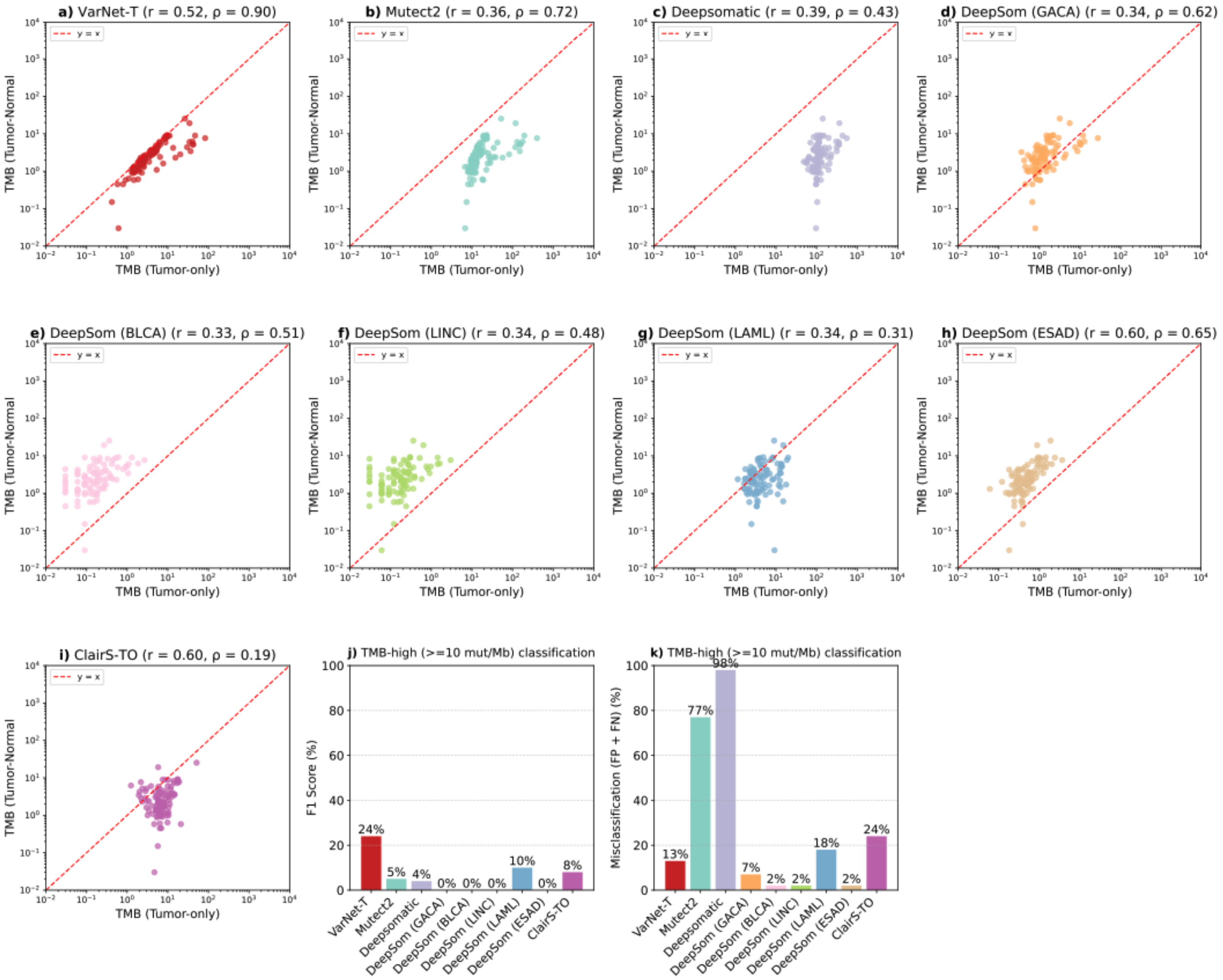

<u>Supplementary Fig. 10:</u> TMB estimated for 100 TCGA WES head and neck tumor samples using tumor-only (x-axis) variant callers compared to tumor-normal variant calls (y-axis). **a** VarNet-T **b** Mutect2 **c** Deepsomatic **d** DeepSom (BLCA) **e** DeepSom (LINC) **f** DeepSom (LAML) **g** DeepSom (GACA) **h** DeepSom (ESAD) **i** ClairS-TO **j** F1 accuracy in classifying TMB-high (≥10) samples **k** Misclassification rates (False Positives + False Negatives) in classifying TMB-high (≥10) samples. Source data are provided as a Source Data file.

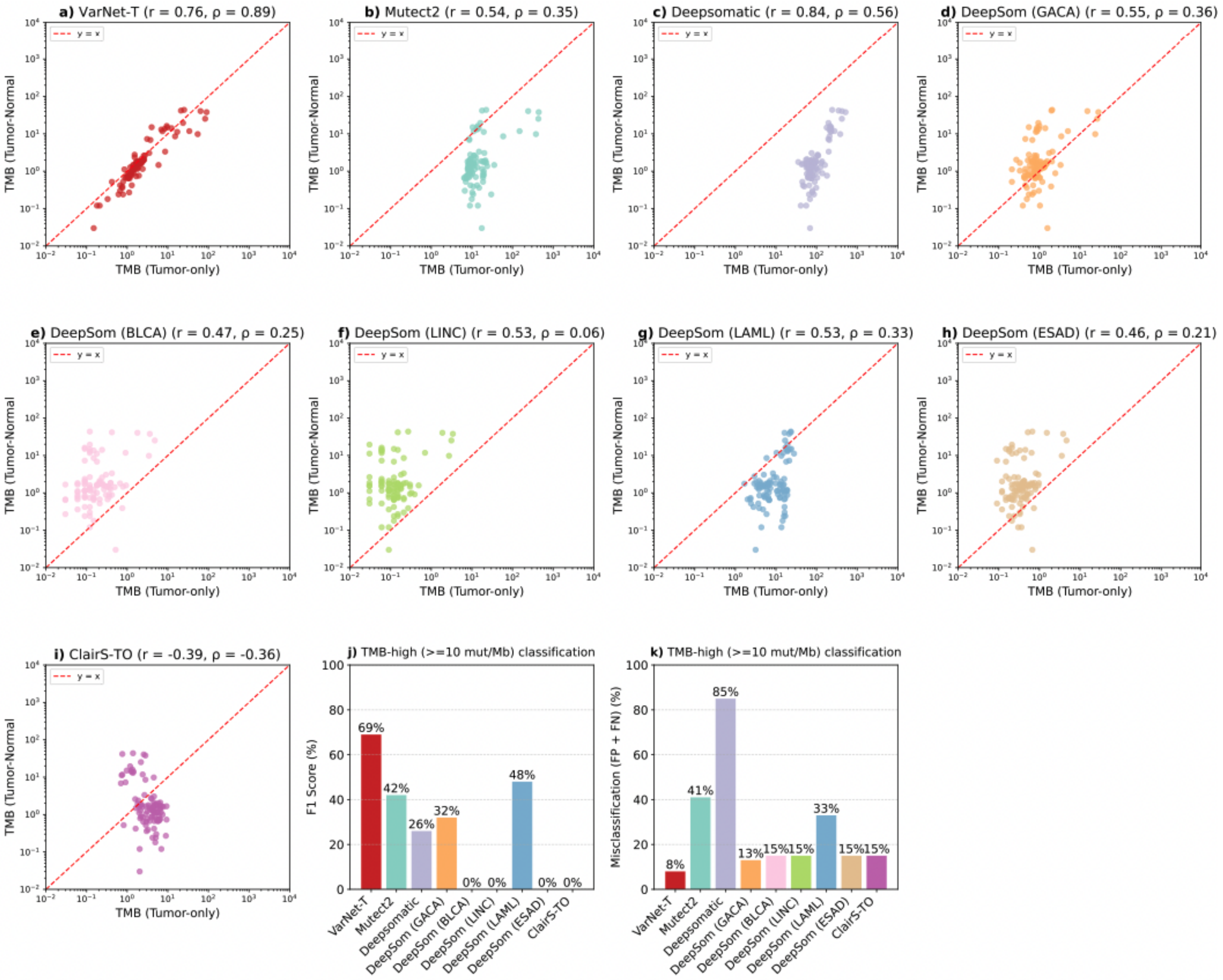

<u>Supplementary Fig. 11:</u> TMB estimated for 100 TCGA WES kidney tumor samples using tumor-only (x-axis) variant callers compared to tumor-normal variant calls (y-axis). a VarNet-T **b** Mutect2 **c** Deepsomatic **d** DeepSom (BLCA) **e** DeepSom (LINC) **f** DeepSom (LAML) **g** DeepSom (GACA) **h** DeepSom (ESAD) **i** ClairS-TO **j** F1 accuracy in classifying TMB-high (≥10) samples **k** Misclassification rates (False Positives + False Negatives) in classifying TMB-high (≥10) samples. Source data are provided as a Source Data file.

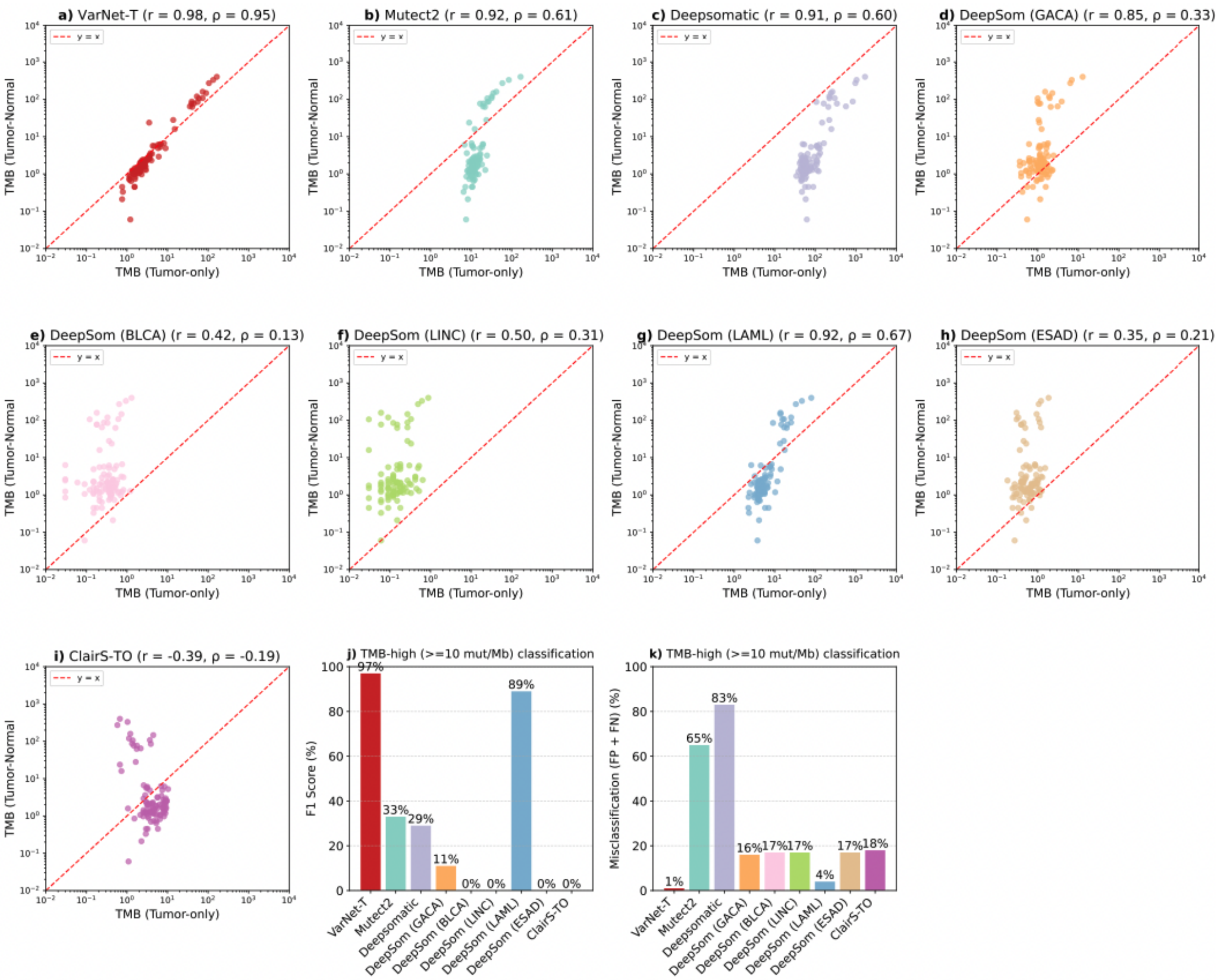

<u>Supplementary Fig. 12:</u> TMB estimated for 100 TCGA WES liver tumor samples using tumor-only (x-axis) variant callers compared to tumor-normal variant calls (y-axis). a VarNet-T **b** Mutect2 **c** Deepsomatic **d** DeepSom (BLCA) **e** DeepSom (LINC) **f** DeepSom (LAML) **g** DeepSom (GACA) **h** DeepSom (ESAD) **i** ClairS-TO **j** F1 accuracy in classifying TMB-high (≥10) samples **k** Misclassification rates (False Positives + False Negatives) in classifying TMB-high (≥10) samples. Source data are provided as a Source Data file.

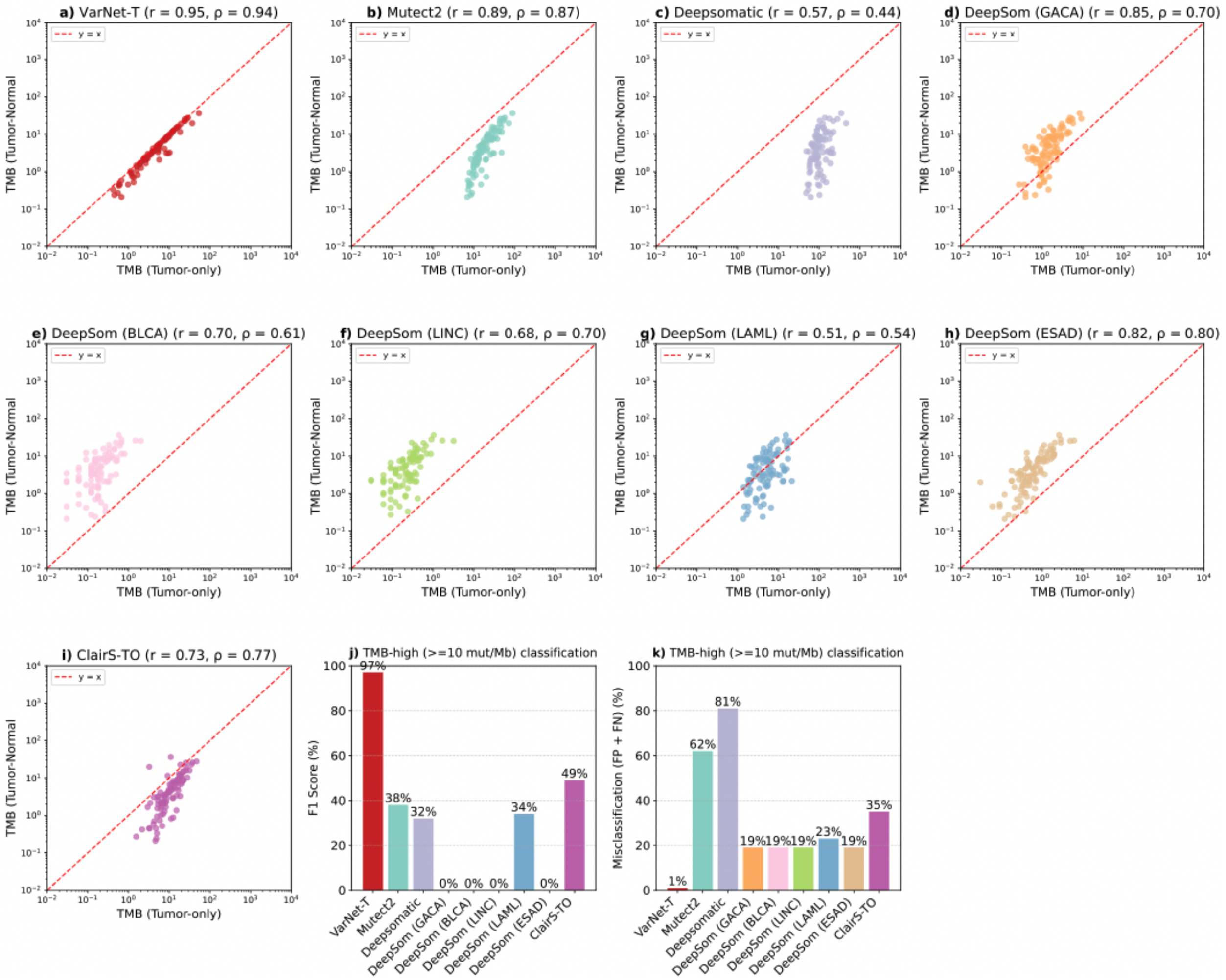

<u>Supplementary Fig. 13:</u> TMB estimated for 100 TCGA WES lung tumor samples using tumor-only (x-axis) variant callers compared to tumor-normal variant calls (y-axis). a VarNet-T **b** Mutect2 **c** Deepsomatic **d** DeepSom (BLCA) **e** DeepSom (LINC) **f** DeepSom (LAML) **g** DeepSom (GACA) **h** DeepSom (ESAD) **i** ClairS-TO **j** F1 accuracy in classifying TMB-high (≥10) samples **k** Misclassification rates (False Positives + False Negatives) in classifying TMB-high (≥10) samples. Source data are provided as a Source Data file.

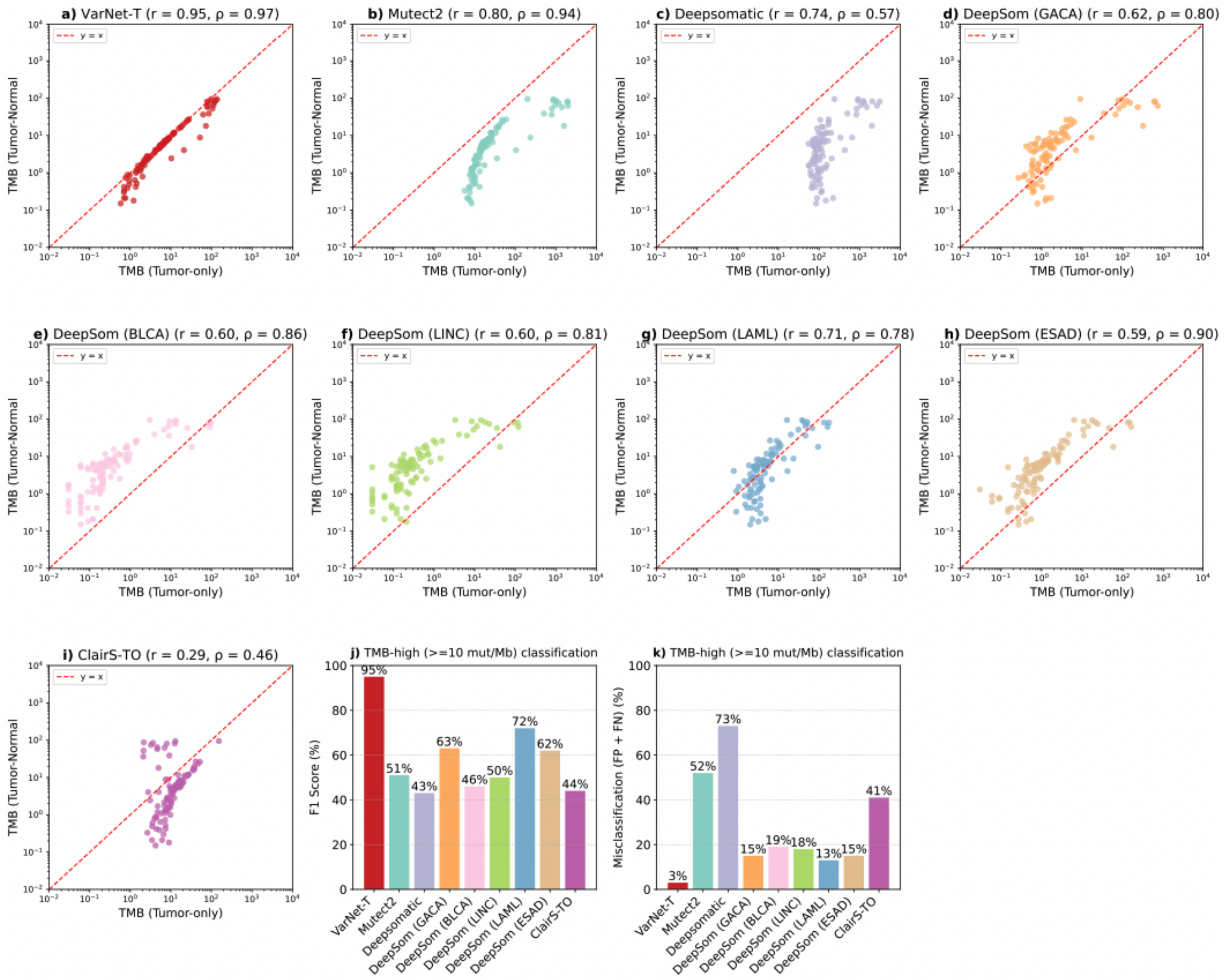

<u>Supplementary Fig. 14:</u> TMB estimated for 100 TCGA WES skin tumor samples using tumor-only (x-axis) variant callers compared to tumor-normal variant calls (y-axis). a VarNet-T **b** Mutect2 **c** Deepsomatic **d** DeepSom (BLCA) **e** DeepSom (LINC) **f** DeepSom (LAML) **g** DeepSom (GACA) **h** DeepSom (ESAD) **i** ClairS-TO **j** F1 accuracy in classifying TMB-high (≥10) samples **k** Misclassification rates (False Positives + False Negatives) in classifying TMB-high (≥10) samples. Source data are provided as a Source Data file.

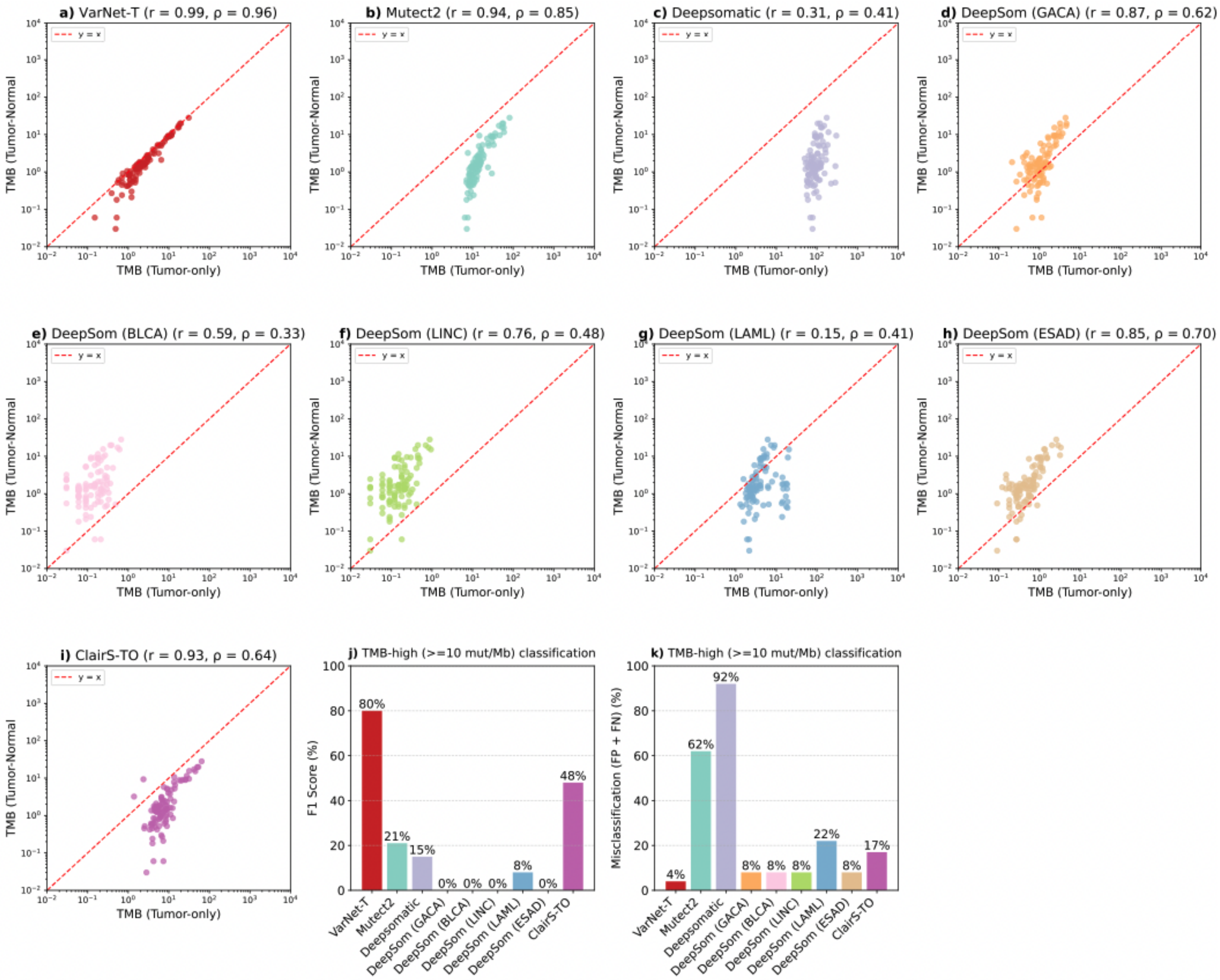

Supplementary Fig. 15: TMB estimated for 100 TCGA WES stomach tumor samples using tumor-only (x-axis) variant callers compared to tumor-normal variant calls (y-axis). a VarNet-T **b** Mutect2 **c** Deepsomatic **d** DeepSom (BLCA) **e** DeepSom (LINC) **f** DeepSom (LAML) **g** DeepSom (GACA) **h** DeepSom (ESAD) **i** ClairS-TO **j** F1 accuracy in classifying TMB-high (≥10) samples **k** Misclassification rates (False Positives + False Negatives) in classifying TMB-high (≥10) samples. Source data are provided as a Source Data file.

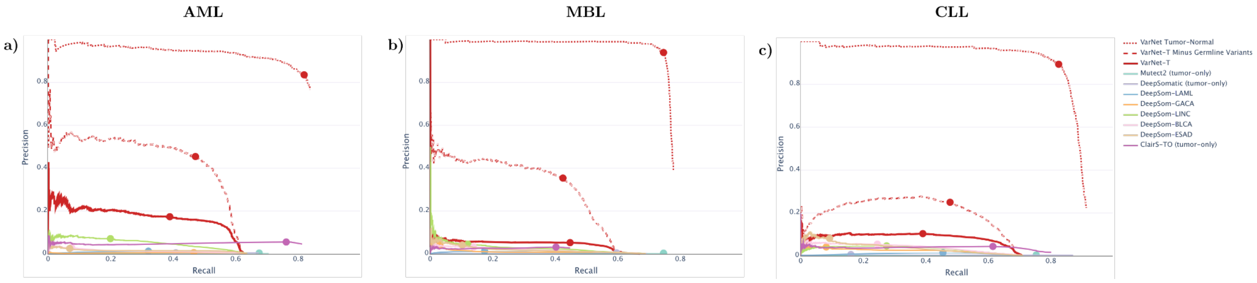

<u>**Supplementary Fig. 16:**</u> **Performance on low mutation burden tumor samples.** PR curves for SNV calling on **a** AML (Acute Myeloid Leukemia) **b** MBL (Medulloblastoma) and **c** CLL (Chronic lymphocytic leukemia).

	SEQC2		COLO829	
**Method**	**SNV**	**INDEL**	**SNV**	**INDEL**
VarNet-T	**0.773**	**0.527**	**0.656**	**0.101**
DeepSom-BLCA	0.540	0.469	0.198	0.029
DeepSom-LINC	0.575	0.462	0.318	0.067
DeepSom-ESAD	0.577	0.363	0.185	0.051
DeepSom-LAML	0.464	0.097	0.146	0.015
DeepSom-GACA	0.246	0.257	0.070	0.016
Deepsomatic (tumor-only)	0.351	0.450	0.118	0.002
Mutect2 (tumor-only)	0.078	0.003	0.072	0.002
ClairS-TO	0.543	0.200	0.270	0.092

Supplementary Table 1: Area under the precision-recall curves (AUPRC) for tumor-only somatic variant calling on the SEQC2 and COLO829 benchmark datasets. The highest AUPRC is highlighted in bold. Source data are provided as a Source Data file.

PCAWG samples	Head and Neck cancer		Stomach cancer		Breast cancer		Bladder cancer		Uterine cancer	
Method/Sample ID	DO15046	DO14566	DO38547	DO38423	DO6144	DO1663	DO804	DO522	DO41398	DO41056
VarNet-T	**0.702**	**0.728**	**0.764**	**0.771**	**0.755**	**0.775**	**0.736**	**0.750**	**0.619**	0.313
Deepsomatic (tumor-only)	0.052	0.200	0.194	0.175	0.220	0.141	0.148	0.104	0.063	0.024
Mutect2 (tumor-only)	0.049	0.202	0.173	0.070	0.148	0.154	0.172	0.126	0.099	0.097
ClairS-TO (tumor-only)	0.318	0.442	0.403	0.479	0.383	0.591	0.381	0.391	0.055	0.139
DeepSom-BLCA	0.423	**0.756**	0.621	0.143	0.718	0.734	0.715	0.706	0.493	0.294
DeepSom-LINC	0.425	0.619	0.600	0.269	0.626	0.612	0.586	0.612	0.398	0.315
DeepSom-ESAD	0.452	0.645	0.674	0.277	0.643	0.704	0.561	0.603	0.543	0.273
DeepSom-LAML	0.097	0.233	0.373	0.112	0.230	0.157	0.249	0.336	0.101	0.079
DeepSom-GACA	0.287	0.674	0.637	0.169	0.450	0.594	0.511	0.663	0.363	**0.417**

Supplementary Table 2: Area under the precision-recall curves (AUPRC) for tumor-only SNV calling on PCAWG tumor only samples using expert-reviewed consensus calls. The highest AUPRC is highlighted in bold. Source data are provided as a Source Data file.

Cancer Type	Number of Samples
Kidney renal papillary cell carcinoma	100
Stomach adenocarcinoma	100
Colon adenocarcinoma / Rectal (CRC)	100
Cervical squamous cell carcinoma and endocervical adenocarcinoma	100
Head and Neck squamous cell carcinoma	100
Skin Cutaneous Melanoma	100
Liver hepatocellular carcinoma	100
Breast invasive carcinoma	100
Lung adenocarcinoma	100
Brain Lower Grade Glioma	100
**Total**	**1,000**

Supplementary Table 3: Cancer type breakdown of TCGA WES samples used for estimating TMB. Source data are provided as a Source Data file.

**Method/Sample**	**AML**	**MBL**	**CLL**
VarNet-T	**0.112**	**0.032**	**0.062**
DeepSom-BLCA	0.010	0.014	0.029
DeepSom-LINC	0.033	0.021	0.024
DeepSom-ESAD	0.011	0.014	0.028
DeepSom-LAML	0.006	0.006	0.007
DeepSom-GACA	0.003	0.008	0.018
Deepsomatic (tumor-only)	0.004	0.003	0.005
Mutect2 (tumor-only)	0.002	0.002	0.002
ClairS-TO	0.040	0.012	0.031

Supplementary Table 4: Area under the precision-recall curves (AUPRC) for tumor-only SNV calling on the AML, and CLL benchmark datasets. The highest AUPRC is highlighted in bold. Source data are provided as a Source Data file.

FEATURE	SNV	INDEL
MIN READ COVERAGE	7	-
MIN VARIANT READS IN TUMOR	2	2
MIN READ MAPPING QUALITY	10	35
MIN VARIANT ALLELE FRACTION	0.035	0.03
MIN BASE QUALITY	22	-

Supplementary Table 5: Filters for whole genome pre-filtering to identify candidate somatic SNVs and indels.These candidates are later subject to germline variant filtering, panel of normal filtering and finally classified by deep learning models. Source data are provided as a Source Data file.

Supplementary Note 1

Impact of Mutational Context on VarNet-T Sensitivity

We calculated VarNet-T's sensitivity for different mutational processes (SBS, Single Base Substitution) using the SEQC2 benchmark dataset. The VarNet-T base model did not display any significant bias towards any one mutational context as it achieved balanced accuracy across all contexts (Supplementary Fig. 17a). However, after applying variant and artifact filtering using gnomAD/dbSNP/Panel-of-Normals, there were some mutational contexts (specifically C>T changes) where sensitivity was lower (Supplementary Fig. 17b).

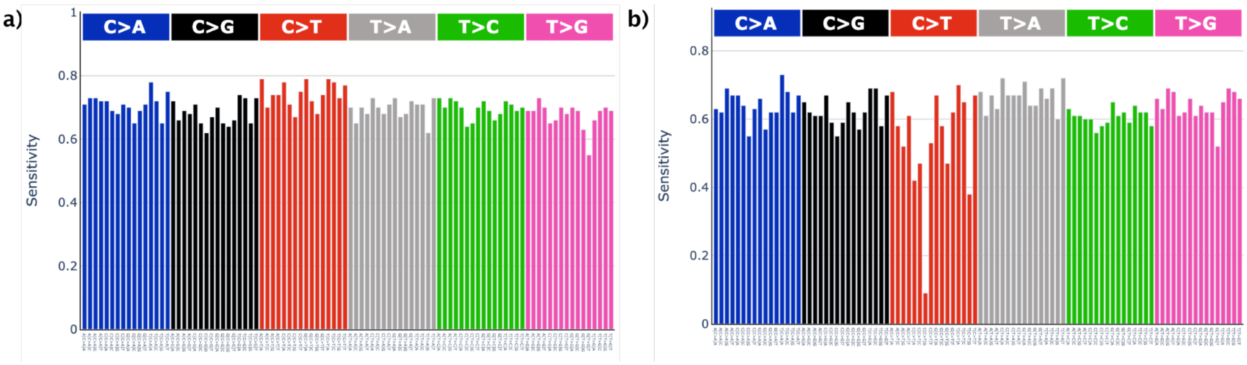

Supplementary Fig. 17: VarNet-T's SNV calling sensitivity across mutational contexts on the SEQC2 sample. a without applying gnomAD-dbSNP-PoN filtering **b** after applying gnomAD-dbSNP-PoN filtering. Source data are provided as a Source Data file.

This is attributable to the fact that this is a common germline as well as artifact signature. Indeed, we measured and plotted the frequency of each mutation context in gnomAD and the 1000 genomes panel of normals and found that C>T variants have the highest proportion in these germline datasets (Supplementary Fig. 18).

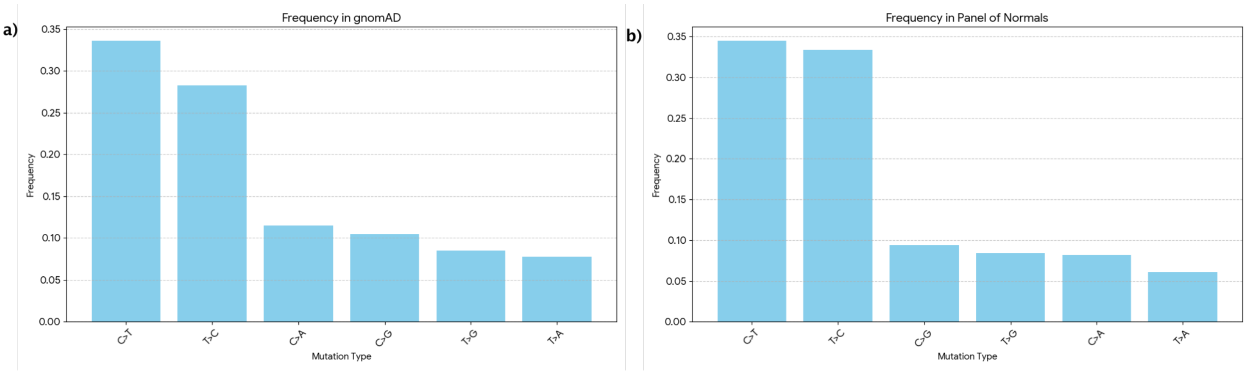

Supplementary Fig. 18: Frequency of SNV base changes observed in **a** gnomAD and **b** 1000 genomes panel of normals. Source data are provided as a Source Data file.

This observed drop in C>T sensitivity is not unique to VarNet-T, as similar performance biases against C>T variants are also seen in other tumor-only methods employing gnomAD filtering, such as Mutect2 and DeepSom (Supplementary Fig. 19).

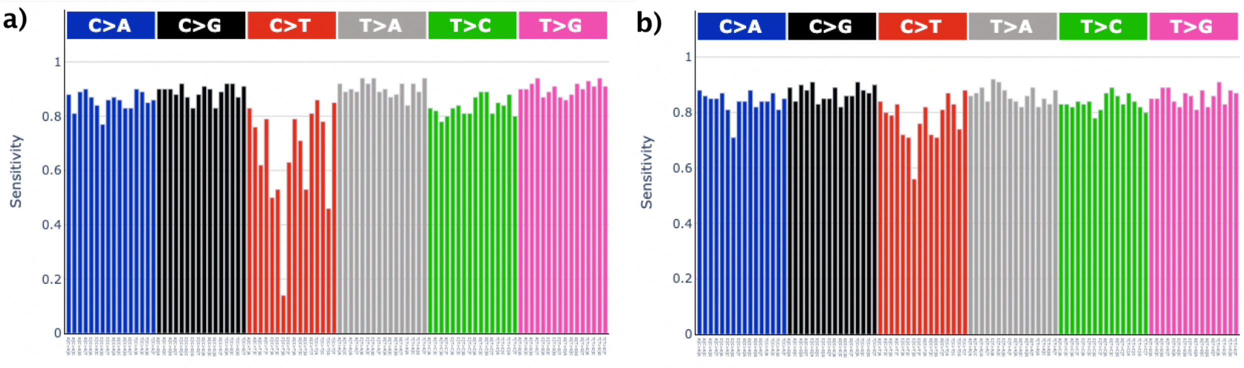

Supplementary Fig. 19: SNV calling sensitivity across mutational contexts on the SEQC2 sample. a DeepSom **b** Mutect2. Source data are provided as a Source Data file.

Supplementary Note 2

Effect of tumor mutation burden on tumor-only variant calling

We hypothesized that samples with low tumor mutation burden are more difficult for tumor-only variant callers due to the challenge of detecting few somatic mutations from a background of many sequencing artifacts and germline variants. To test this hypothesis, we created a series of samples with increasing tumor mutation burden. Starting with the SEQC2 sample, which contains 39,447 SNVs, we generated a series of samples with decreasing mutation burdens by randomly excluding subsets of SNVs from the ground-truth set from the evaluation. These samples were created to contain 1,000, 2,000, ..., up to the full set of SNVs. We observed that accuracy of VarNet-T increased steadily with mutation burden (Supplementary Fig. 20). The performance improvement is mainly due to improved precision rather than sensitivity as we observe that the maximum sensitivity is the same in each curve. This reflects that tumor-only variant calling is sensitive to tumor mutation burden and could be more accurate for samples with high tumor mutation burden.

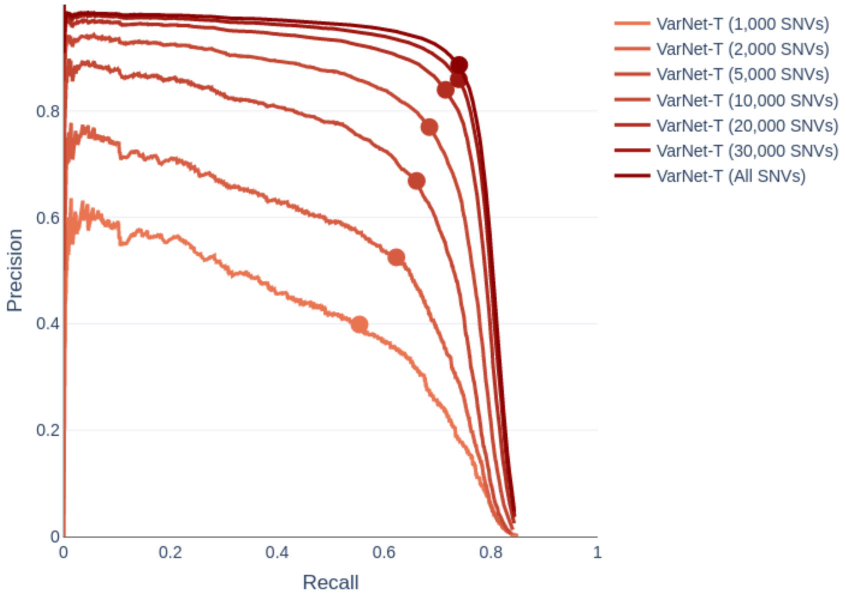

<u>Supplementary Fig. 20:</u> Precision-recall curves of tumor-only SNV calling on SEQC2 with varying mutation burden.

Supplementary Note 3

Shared sequencing artifacts between matched tumor and normal samples

We hypothesized that a significant proportion of sequencing artifacts observed in tumor samples are also present in the matched normal samples. This overlap allows tumor-normal variant callers to identify low-frequency sequencing artifacts occurring in both tumor and normal samples. To test this hypothesis, we extracted all SNV variant candidates (VAF > 3.5%) from the matched normal and tumor samples of CLL and MBL individually. A germline variant caller was then applied to each matched normal sample, and germline variants were removed from the variant candidates in both the normal and tumor samples for the corresponding pairs. Additionally, ground-truth somatic SNVs were excluded from the variant lists.

The remaining variants in each normal and tumor sample are presumed to predominantly represent sequencing artifacts, as germline and somatic events had been filtered out. In the CLL sample, we identified 1,525,250 and 1,352,545 putative sequencing artifact variants in the matched normal and tumor samples, respectively. Of these, 939,379 variants were shared between the matched normal and tumor samples, indicating that 70% of the sequencing artifact variants observed in the tumor sample were also present in the matched normal sample. As expected, the VAF distribution of these shared sequencing artifacts was lower than that of shared germline mutations (Supplementary Fig. 21). These findings support our hypothesis that the matched normal sample can aid in filtering shared sequencing artifacts in the tumor sample.

A similar pattern was observed in the MBL sample, where 64% of sequencing artifacts detected in the tumor sample were also found in the matched normal sample.

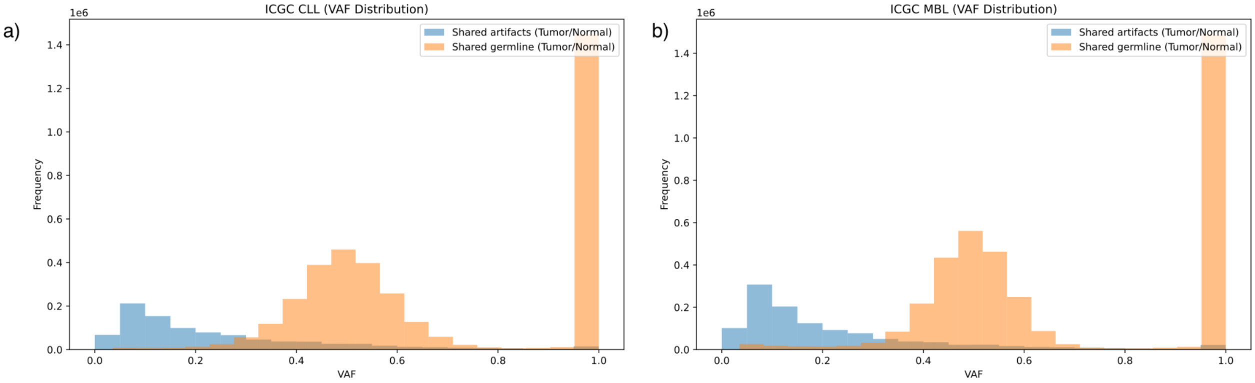

Supplementary Fig. 21: VAF distribution of shared sequencing artifacts and germline mutations between matched normal and tumor samples. a ICGC CLL sample, **b** ICGC MBL sample.

We further performed a post-hoc analysis on these shared artifact sites with respect to common challenging regions. While we observed that a portion of the shared, putatively artifactual sites did fall within repeat regions (as defined by RepeatMasker: https://genome.ucsc.edu/cgi-bin/hgTrackUi?g=rmsk), we found that applying a strict filter on these regions is highly detrimental to sensitivity. Specifically, in the CLL sample, 664 out of 1,319 true somatic SNVs were also located within these repeat regions. This demonstrates a significant overlap where

over 50% of ground truth mutations are situated in these regions for this CLL sample. This critical overlap means that filtering based purely on repeat region annotations would result in an unacceptable level of false negatives. This finding highlights the necessity of a machine learning model, like VarNet-T, to accurately distinguish true somatic mutations from systemic sequencing noise in these challenging genomic areas. We also found that only <15% of these shared sites had a mean coverage below 30x. This indicates that these artifacts are not simply confined to regions of critically low sequencing depth. While established public blacklists might filter a small subset of the ~1 million shared artifactual variants, simple post-hoc metrics like coverage or repeat region annotation may be ineffective or negatively affect accuracy. Hence, these shared sites may represent complex, systemic sequencing noise that is not reducible to simple genomic filters. This presents a challenge for all tumor-only variant callers.

Supplementary Note 4

Impact of Training Variant Distribution on VarNet-T Performance

We trained two models to reflect a different distribution of somatic vs non-somatic variants by upweighting non-somatic variants during training. We trained one model to reflect a ~33% somatic vs ~66% non-somatic variants and another model with ~20% somatic vs ~80% non-somatic variants. Both these models performed worse than our baseline VarNet-T model (~50% somatic vs ~50% non-somatic variants) on the low TMB ICGC CLL/MBL benchmark samples (Supplementary Table 6). These results suggest that the difficulty is primarily in distinguishing artifacts in the absence of a matched normal sample.

Model	MBL (AUPRC)	CLL (AUPRC)
VarNet-T (50% somatic vs 50% non-somatic)	0.032	0.062
VarNet-T (~33% somatic vs ~66% non-somatic)	0.029	0.060
VarNet-T (20% somatic vs 80% non-somatic)	0.027	0.056

Supplementary Table 6: Performance of VarNet-T trained on different proportions of somatic vs non-somatic variants on low mutation burden tumors. Source data are provided as a Source Data file.
