# Supplementary material for: Improved tumor-only variant calling and mutation burden estimation with VarNet-T
Source: Nat Commun. 2026 Apr 9;17:5019. doi: 10.1038/s41467-026-71705-4 (PMC13243554; doi:10.1038/s41467-026-71705-4)
Supplement: Supplementary file 3 — Description of Additional Supplementary Files [file 41467_2026_71705_MOESM3_ESM.pdf]

Description of Additional Supplementary Files

Supplementary Data 1: TCGA_samples.xlsx

This file contains the list of TCGA sample ids used for TMB evaluation (1000 WES samples) and also the TCGA sample ids used in the training cohort. These do not overlap.
