# Supplementary material for: Improved tumor-only variant calling and mutation burden estimation with VarNet-T
Source: Nat Commun. 2026 Apr 9;17:5019. doi: 10.1038/s41467-026-71705-4 (PMC13243554; doi:10.1038/s41467-026-71705-4)
Supplement: Supplementary file 2 — Peer Review File [file 41467_2026_71705_MOESM2_ESM.pdf]

nature portfolio

Peer Review File

Improved tumor-only variant calling and mutation burden estimation with VarNet-T

Corresponding Author: Dr Anders Jacobsen Skanderup

This file contains all reviewer reports in order by version, followed by all author rebuttals in order by version.

Version 1:

Reviewer comments:

Reviewer #1

(Remarks to the Author)
Krishnamachari et al. present VarNet-T, a novel weakly supervised deep learning model to detect somatic mutations for tumour sequencing only (in the absence of a matched normal sample). VarNet-T is an extension of VarNet (Krishnamachari et al. Nat Comms 2022) to meet the challenges of detecting somatic mutations when only tumour sequencing is available. This is particularly important as sequencing is increasingly being used in clinical settings to measure genomic biomarkers, such as tumour mutation burden, and matched normal sequencing is not always available. VarNet-T was trained on over 300 tumour whole genomes across seven cancer types leveraging 1.25 million somatic SNVs and 1.05 million somatic INDELs. The authors demonstrate VarNet-T outperforms (based on precision and recall) other commonly used tumour-only somatic mutation calling tools, such as Mutect2 and DeepSomatic, when run on two cell line benchmarking datasets, SEQC2 and TGEN, as well as three tumour samples from ICGC, CLL, MBL and AML. VarNet-T performs better on tumours with high mutation burden than low mutation burden and more accurately identifies TMB-high tumours across 1,000 WXS tumour samples.

VarNet-T addresses an important challenge not only in cancer genomics research but also for the clinical translation of genomic biomarkers. The data is clearly presented and the manuscript well written. Much of the benchmarking presented focuses on two cell lines with high mutation burden (SEQC2 and COLO829) or TCGA samples which appear were also used to train the model. Only Supplementary Figure 3 shows performance on tumour samples not included in model training for which VarNet-T has low performance (although still better than other models evaluated). It would be helpful to see the performance of VarNet-T on additional tumour samples not included in training, such as non-TCGA samples from PCAWG that have consensus somatic mutation calls from tumour/normal matched sequencing. It would also be of interest how the performance of VarNet-T varies by mutational process (given the importance of mutational sequence context; Figure 6) and by ancestry. Some additional methodological points require clarification (see below). More specific comments are provided below.

Major:
1. The majority of benchmarking of VarNet-T shown in the main figures is on SEQC2 and TGEN. While these are established reference datasets, they are based on cell lines whose mutational patterns do not always reflect those observed in tumours. For example, the TNBC cell line used in SEQC2 displays BRCAness and the COLO829 cell line is a metastatic line leading to the higher mutation burden observed in these samples. Thus, the mutational patterns in these samples do not reflect those observed in untreated primary tumours. The authors also show performance in three tumour samples with lower mutation burden: CLL, MBL and AML tumours from ICGC. Here, VarNet-T performs substantially worse (AUPRC = 0.062-0.112). The authors report this is due to the lower mutational burden, however, the mutational burden observed in these tumours is more aligned with expected mutational burden for many tumour types. Are the authors proposing VarNet-T should only be used for specific tumour types with sufficiently high mutation burden. Can the authors also confirm that VarNet-T will perform better in higher mutation burden tumour samples (not cell line derived) leveraging sequencing data from non-TCGA samples from PCAWG (PMID: 32025007) that has expert-reviewed consensus calls?
2. Similarly, the authors show that increasing TMB increases the accuracy of VarNet-T due to increased precision. Recall remains consistent across TMB. The increase in precision may imply that VarNet-T calls a similar number of variants across all samples and thus overcalling in low TMB tumours (low precision). Is this indeed the case? Could this be a consequence of the fact that the training data included ~50% somatic vs ~50% non-somatic variants, a more balanced distribution than expected in real data? Would changing this distribution in the training data improve VarNet-T performance in low TMB

tumours? Would training separate models designed for low TMB and high TMB tumours help overcome this issue?
3. The distribution of cancer types used to train VarNet-T (Fig 1a) is heavily skewed towards colon cancer. Is VarNet-T better at detecting mutational processes that are enriched in colon cancer vs others? Is there any relationship between the mutational processes present in each tumour and accuracy of mutation calls? This is particularly called into question in light of Fig 6 which shows the emphasis the model places on the sequence context of the mutation.
4. VarNet-T prefilters variants prior to classification using a germline filter. The methods indicate this is done using gnoMAD and common variants in dbSNP. Was all of gnoMAD considered as there are number of rare variants in gnoMAD that have the potential to be somatic mutations in other individuals? Where these also filtered out? Could the authors please clarify.
5. One concern of tumour-only somatic mutation (SNV and INDEL) detection, particularly with respect to TMB determination, is that germline contamination differences across ancestries, in part, due to reference bias (PMID: 36179682). How does performance of VarNet-T differ by ancestry? Was ancestry considered in the selection of 100 TCGA tumours per cancer type when evaluating TMB (Fig 4)?
6. When subsampling SEQC2 to create in silico tumours with varying TMB, were variants subsampled according to VAF?
7. Given TCGA was used to train VarNet-T. Can it be reliably used to evaluate tumour-only TMB measures in TCGA?
8. The authors suggest that matched tumour and normal sequencing allows models to learn shared patterns in sequencing artifacts. They report over 1 million sequencing artifacts in identified in both tumour and normal sequencing from an individual with CLL. Could the authors comment on other characteristics of these putative sequencing artifacts? Are they occurring in repeat regions? At regions of low coverage? Could post-hoc filters aid in removing these variants?

Minor comments:
1. Supplementary figures references are out of order and can be confusing to follow at times.
2. In Methods, under Training Data, VarNet is cited as [1] which does not refer to the VarNet citation
3. In the Methods, the authors cite the VarNet manuscript for more details on the data that was used to train VarNet-T. The training data is an important consideration when assessing the model, thus, it is requested that the author restate the training data directly in the Methods of this manuscript.

(Remarks on code availability)
Tried to run docker as outlined in README file, however, the first filter step (filter.py) failed with the error: "filter.py: error: the following arguments are required: --normal_bam". This is likely a small bug with how the command line arguments were set and would just require an update.

Reviewer #2

(Remarks to the Author)
The difference between VarNet-T is quite substantial, yet there isn't a clear explaination as to what specific innovations are responsible for this difference. Deepsomatic, for example, is a conceptually similar application.

Was the subtraction of germline variants (via gnomAD) done with the same variants across all tumor-only callers? The methods only mention this being done for VarNet-T.

If the germline pre-filtering procedure (Supplemental Table 3) is applied to VCFs from other tumor-only methods do their metrics improve substantially?

217-228: The improvement represented by VarNet-T in estimating TMB is substantial, requiring an explaination for the improvement over other methods. What mutation tumour/normal mutation caller was used for TMB estimation in figure 4 and supplemental figs 8-17? Are these results sensitive to the choice of tumor-normal variant caller?

For other ML-based tools (e.g. deepvariant) is there an indication of how many examples went into training the model vs the number that went into VarNet-T?

Even when carried out with the best of intentions, benchmarking by the authors will naturally be biased towards the tool the authors are presenting given greater familiarity around parameterisation. Would it be possible for a comparison of results previously obtained by authors of a leading competing method?

Software should be released on github with more extensive instructions on its use.

(Remarks on code availability)
This needs to be made more accessible e.g. when posted to github, instructions need to include more examples around different use cases and detailed installation instructions in addition to test data to verify a users's installation. The code itself includes hard-coded paths local to the authors and other artefacts that should be cleaned up prior to release.

Version 2:

Reviewer comments:

Reviewer #1

(Remarks to the Author)

I thank the reviewers for their thoughtful responses to my comments. My comments have largely been addressed. I have only two minor remaining items:

1. In response to a few comments, it appears that the new data was only included in the response to the reviewer not in the manuscript itself. I believe these analyses/data (even if not beneficial to model performance) would still be of interest to readers and support the parameterization choices made. Could the authors add the following to the manuscript in supplementary:
a. Could the authors please incorporate the data showing the sensitivity by mutational process into the supplementary of the manuscript (Comment 3)?
b. Similarly, could the authors add the model performance when trained with different proportions of somatic and non-somatic variants to the manuscript (Comment 2)?
c. Finally, could the authors include a statement about the limitations (i.e. the reduction in sensitivity) of post-hoc genomic filters in the manuscript (Comment 8)?

2. Regarding the use of TCGA in the training data, I appreciate the authors only considered a subset of tumours with WGS in training leaving 1,000 WXS samples for testing. However, some donors in TCGA have both WXS and WGS profiling. Could the authors please confirm that no donors overlap their training and testing cohorts and include a statement in the manuscript?

(Remarks on code availability)
Previous bug has been fixed.

Reviewer #2

(Remarks to the Author)
I thank the authors for their comprehensive consideration and response to my concerns - I am satisfied with this response.

(Remarks on code availability)
The operation of VarNet-T within VarNet by omitting a normal sample has been clarified.

Open Access This Peer Review File is licensed under a Creative Commons Attribution 4.0 International License, which permits use, sharing, adaptation, distribution and reproduction in any medium or format, as long as you give appropriate credit to the original author(s) and the source, provide a link to the Creative Commons license, and indicate if changes were made.
In cases where reviewers are anonymous, credit should be given to 'Anonymous Referee' and the source.
The images or other third party material in this Peer Review File are included in the article's Creative Commons license, unless indicated otherwise in a credit line to the material. If material is not included in the article's Creative Commons license and your intended use is not permitted by statutory regulation or exceeds the permitted use, you will need to obtain permission directly from the copyright holder.
To view a copy of this license, visit https://creativecommons.org/licenses/by/4.0/

We thank the reviewers for their feedback and constructive comments, which have significantly improved the manuscript. We have made point-by-point responses to all the reviewers' comments and descriptions of the corresponding changes to the manuscript below. All modified text and figures in the manuscript and supplementary sections are in blue for ease of review.

We are also pleased to report that **VarNet-T significantly outperformed the recently published ClairS-TO short-read model (**Nature Communications: **https://www.nature.com/articles/s41467-025-64547-z)** in all our primary benchmarks (updated Figs. 2,3,4,5).

Reviewer 1 Comments

Reviewer 1, General Comments

Reviewer Comment	*Krishnamachari et al. present VarNet-T, a novel weakly supervised deep learning model to detect somatic mutations for tumour sequencing only (in the absence of a matched normal sample). VarNet-T is an extension of VarNet (Krishnamachari et al. Nat Comms 2022) to meet the challenges of detecting somatic mutations when only tumour sequencing is available. This is particularly important as sequencing is increasingly being used in clinical settings to measure genomic biomarkers, such as tumour mutation burden, and matched normal sequencing is not always available. VarNet-T was trained on over 300 tumour whole genomes across seven cancer types leveraging 1.25 million somatic SNVs and 1.05 million somatic INDELs. The authors demonstrate VarNet-T outperforms (based on precision and recall) other commonly used tumour-only somatic mutation calling tools, such as Mutect2 and DeepSomatic, when run on two cell line benchmarking datasets, SEQC2 and TGEN, as well as three tumour samples from ICGC, CLL, MBL and AML. VarNet-T performs better on tumours with high mutation burden than low mutation burden and more accurately identifies TMB-high tumours across 1,000 WXS tumour samples.*<br>*VarNet-T addresses an important challenge not only in cancer genomics research but also for the clinical translation of genomic biomarkers. The data is clearly presented and the manuscript well written. Much of the benchmarking presented focuses on two cell lines with high mutation burden (SEQC2 and COLO829) or TCGA samples which appear were also used to train the model. Only Supplementary Figure 3 shows performance on tumour samples not included in model training for which VarNet-T has low performance (although still better than other models evaluated). It would be helpful to see the performance of VarNet-T on additional tumour samples not included in training, such as non-TCGA samples from PCAWG that have consensus somatic mutation calls from tumour/normal matched sequencing. It would also be of interest how the performance of VarNet-T varies by mutational process (given the importance of mutational sequence context; Figure 6) and by ancestry. Some additional methodological points require clarification (see below). More specific comments are provided below.*
Author Response	We thank the reviewer for the positive feedback and suggestions to improve the manuscript. Below we provide a detailed point-by-point response for all the comments.

Reviewer 1, Major Comment 1

Reviewer Comment	*1. The majority of benchmarking of VarNet-T shown in the main figures is on SEQC2 and TGEN. While these are established reference datasets, they are based on cell lines whose mutational patterns do not always reflect those observed in tumours. For example, the TNBC cell line used in SEQC2 displays BRCAness and the COLO829 cell line is a metastatic line leading to the higher mutation burden observed in these samples. Thus, the mutational patterns in these samples do not reflect those observed in untreated primary tumours. The authors also show performance in three tumour samples with lower mutation burden: CLL, MBL and AML tumours from ICGC. Here, VarNet-T performs substantially worse (AUPRC = 0.062-0.112). The authors report this is due to the lower mutational burden, however, the mutational burden observed in these tumours is more aligned with expected mutational burden for many tumour types. Are the authors proposing VarNet-T should only be used for specific tumour types with sufficiently high mutation burden. Can the authors also confirm that VarNet-T will perform better in higher mutation burden tumour samples (not cell line derived) leveraging sequencing data from non-TCGA samples from PCAWG (PMID: 32025007) that has expert-reviewed consensus calls?*

Author Response	Thanks for the suggestion. We further benchmarked tumor-only SNV calling accuracy using WGS samples from the PCAWG (Pan-cancer analysis of whole genomes) consortium[18], which contains WGS data for a large number of tumor samples across multiple cancer types. For our benchmark, we first selected five solid cancer types not used during training and then randomly selected two samples per cancer type derived from primary tumors with high somatic mutation burden (10k-100k SNVs per sample). These ten samples represented five different cancer types: breast, bladder, stomach adenocarcinoma, head and neck squamous cell carcinoma, and uterine cancer. Importantly, the samples and cancer types were not included in VarNet-T's training cohorts and hence tested VarNet-T's generalization ability. We used expert reviewed consensus variant calls provided by the PCAWG consortium as ground-truth sets for evaluation. VarNet-T substantially outperformed other callers (Fig. 3) achieving an average AUPRC of 0.69, performing better than Mutect2 (0.13), Deepsomatic (0.13), ClairS-TO (0.36) and all DeepSom models (DeepSom-BLCA (0.56), DeepSom-LINC (0.51), DeepSom-ESAD (0.54), DeepSom-LAML (0.20), DeepSom-GACA (0.48)). AUPRCs of each method per sample are listed in Suppl. Table 2. These results demonstrate VarNet-T's generalization ability for tumor-only whole genome variant calling. Notably, while DeepSom's performance was highly variable across its models - with the worst scoring 0.20 and the best 0.56 - VarNet-T's single pan-cancer model provided better performance, eliminating the need for uncertain, cancer-specific model selection. VarNet-T also significantly outperformed other deep learning methods, Deepsomatic and ClairS-TO, on these real tumor samples. Unlike these competing methods, which were trained only on synthetic mutations or mutations derived from cancer cell lines, VarNet-T is trained on a large number of real tumor-derived mutations using weak supervision, allowing it to capture the complex biological variation, tumor heterogeneity and normal cell contamination that cell lines lack.

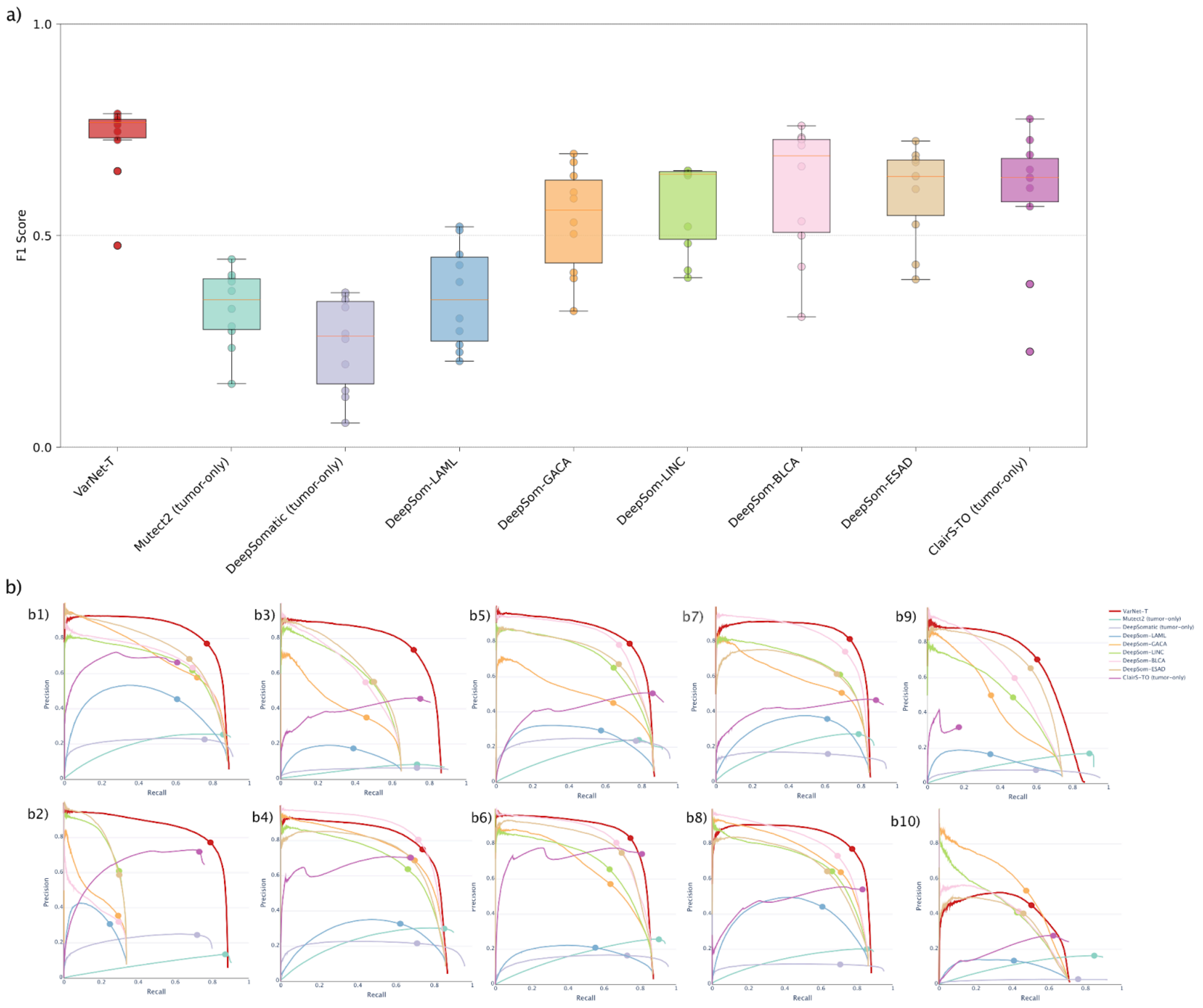

Fig. 3 Tumor-only variant calling performance on PCAWG WGS tumor samples. **a** F1 accuracy for tumor-only SNV calling on PCAWG WGS tumor samples**.** Box plots of the best F1 scores achieved for tumor-only SNV calling across ten PCAWG WGS samples comprising five cancer types. VarNet-T achieves the highest F1 scores with least variance compared to other callers. **b** Precision-recall curves for tumor-only SNV calling on PCAWG tumor samples. Stomach Adenocarcinoma: **b1** DO38547 **b2** DO38423; Head and neck squamous cell carcinoma: **b3** DO15046 **b4** DO14566, Breast cancer: **b5** DO6144 **b6** DO1663; Bladder cancer **b7** DO804 **b8** DO522; Uterine cancer: **b9** DO41398 **b10** DO41056. Source data is provided as a Source Data file. Source data is provided as a Source Data file.

Edits to manuscript	We have added these new results to the revised manuscript. See new results section entitled **Benchmarking using whole cancer genomes from PCAWG**

Reviewer 1, Major Comment 2

Reviewer Comment	*2. Similarly, the authors show that increasing TMB increases the accuracy of VarNet-T due to increased precision. Recall remains consistent across TMB. The increase in precision may imply that VarNet-T calls a similar number of variants across all samples and thus overcalling in low TMB tumours (low precision). Is this indeed the case? Could this be a consequence of the fact that the training data included ~50% somatic vs ~50% non-somatic variants, a more balanced distribution than expected in real data? Would changing this distribution in the training data improve*

VarNet-T performance in low TMB tumours? Would training separate models designed for low TMB and high TMB tumours help overcome this issue?

Author Response

Thanks for the insightful question and suggestion. To address the reviewers question, we trained two models to reflect a different distribution of somatic vs non-somatic variants by upweighting non-somatic variants during training. We trained one model to reflect a ~33% somatic vs ~66% non-somatic variants and another model with ~20% somatic vs ~80% non-somatic variants. Both these models performed worse than our baseline VarNet-T model (~50% somatic vs ~50% non-somatic variants) on the low TMB samples CLL/MBL. We report the AURPCS below. These results suggest that the difficulty is primarily in distinguishing artifacts in the absence of a matched normal sample.

Model	MBL (AUPRC)	CLL (AUPRC)
VarNet-T (50% somatic vs 50% non-somatic)	0.032	0.062
VarNet-T (~33% somatic vs ~66% non-somatic)	0.029	0.060
VarNet-T (20% somatic vs 80% non-somatic)	0.027	0.056

Reviewer 1, Major Comment 3

Reviewer Comment

3. The distribution of cancer types used to train VarNet-T (Fig 1a) is heavily skewed towards colon cancer. Is VarNet-T better at detecting mutational processes that are enriched in colon cancer vs others? Is there any relationship between the mutational processes present in each tumour and accuracy of mutation calls? This is particularly called into question in light of Fig 6 which shows the emphasis the model places on the sequence context of the mutation.

Author Response

Thanks for the insightful question. We calculated VarNet-T's sensitivity for different mutational processes (SBS, Single Base Substitution) using the SEQC2 benchmark dataset. The VarNet-T model did not display any significant bias towards any one mutational context as it achieved balanced accuracy across all contexts (figure below).

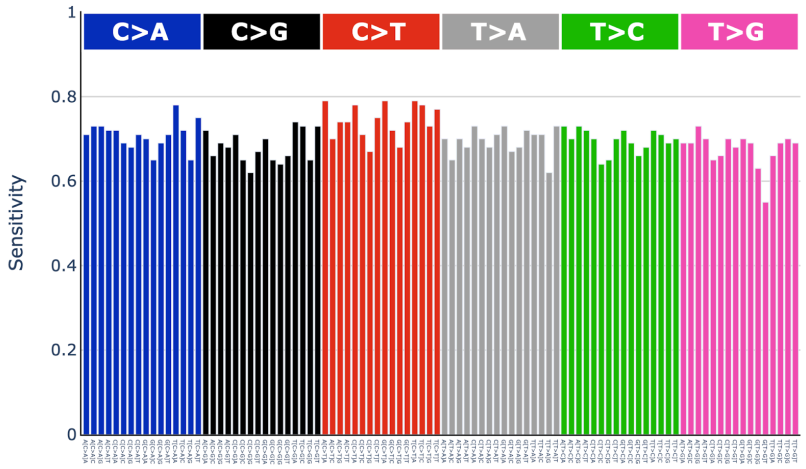

Figure: VarNet-T model accuracy per mutation context.

However, after applying variant and artifact filtering using gnomAD/dbSNP/Panel-of-Normals, there were some mutational contexts (specifically C>T changes) where sensitivity was lower (see figure below).

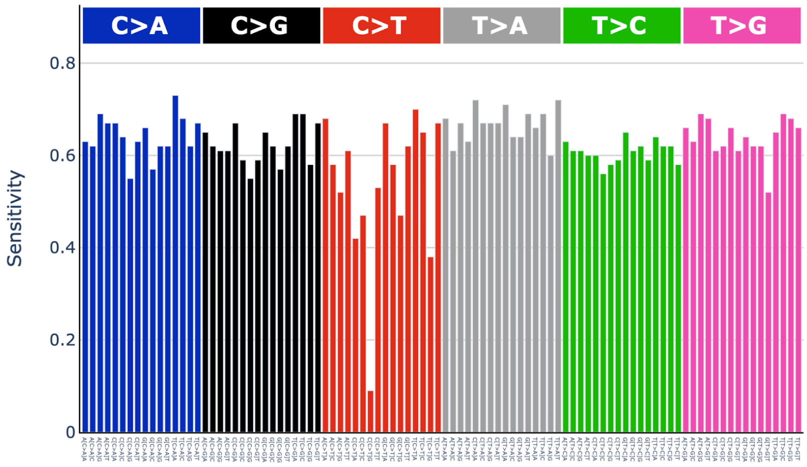

Figure: VarNet-T sensitivity <u>after</u> applying gnomAD/dbSNP/PoN filtering:

This is attributable to the fact that this is a common germline as well as artifact signature. Indeed, we measured and plotted the frequency of each mutation context in gnomAD and the 1000 genomes panel of normals and found that C>T variants have the highest proportion in these germline datasets (figures below).

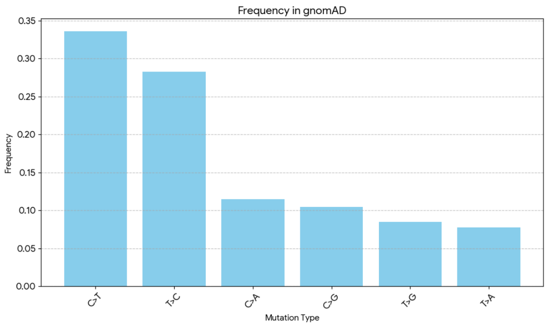

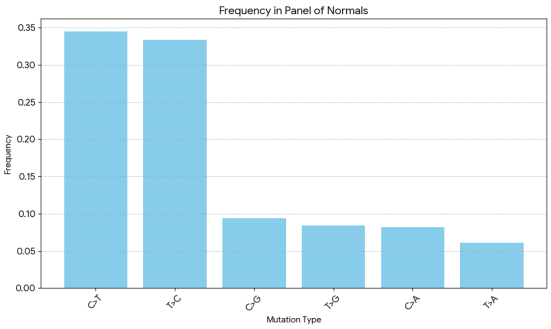

This observed drop in C>T sensitivity is not unique to VarNet-T, as similar performance biases against C>T variants are also seen in other tumor-only methods employing gnomAD filtering, such as Mutect2 and DeepSom (see figures below).

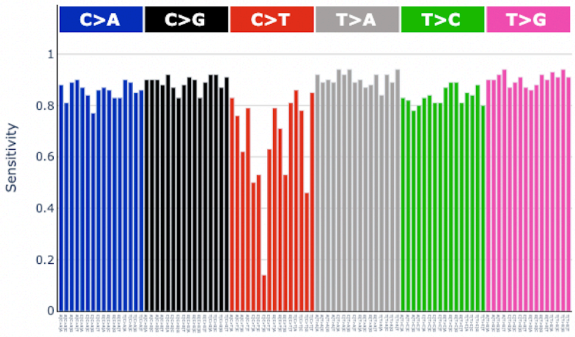

DeepSom

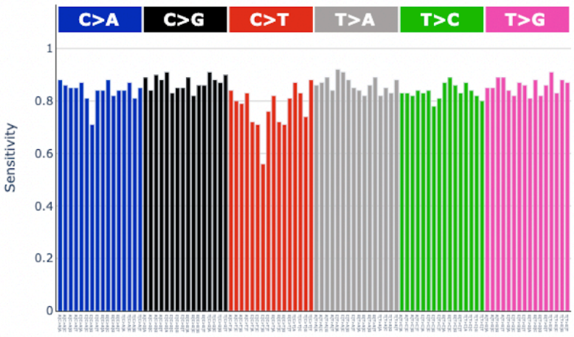

Mutect2

Reviewer 1, Major Comment 4

Reviewer Comment	*4. VarNet-T prefilters variants prior to classification using a germline filter. The methods indicate this is done using gnoMAD and common variants in dbSNP. Was all of gnoMAD considered as there are number of rare variants in gnoMAD that have the potential to be somatic mutations in other individuals? Where these also filtered out? Could the authors please clarify.*
Author Response	Thanks for the question. We considered all gnomAD variants as this yielded better overall trade-off between sensitivity and precision. Although a small fraction of variants classified as germline in gnomAD may be somatic in individual cases, these events are rare, and established germline oncogenic mutations can be recovered via positive filtering (user option in VarNet-T software). In the dbSNP database, limiting to COMMON variants (with population AF>1%) lead to a better trade-off as this applied in addition to the gnomAD filtering.
Edits to software	We have added a –whitelist_vcf option to the software to whitelist any known variants that may otherwise be filtered due to their presence in gnomAD or dbSNP

Reviewer 1, Major Comment 5

Reviewer Comment	*5. One concern of tumour-only somatic mutation (SNV and INDEL) detection, particularly with respect to TMB determination, is that germline contamination differences across ancestries, in part, due to reference bias (PMID: 36179682). How does performance of VarNet-T differ by ancestry? Was ancestry considered in the selection of 100 TCGA tumours per cancer type when evaluating TMB (Fig 4)?*
Author Response	Thank you for the suggestion. We analyzed Tumor Mutational Burden (TMB) estimation accuracy by ancestry within the TCGA cohort, utilizing ancestry annotations from the linked study (https://gdc.cancer.gov/about-data/publications/CCG-AIM-2020). We limited to samples which had available ancestry annotations (N=784). Our evaluation of TMB estimation accuracy (specifically for TMB-high, defined as >10 mut/MB) across European, African, Asian, and Native American ancestries revealed a key finding: TMB-high estimation accuracy was superior for samples with European ancestry**.** As the reviewer suggested, this observed discrepancy is likely contributed to by ancestry-related reference bias in the current variant calling pipelines and germline mutation databases such as gnomAD.

	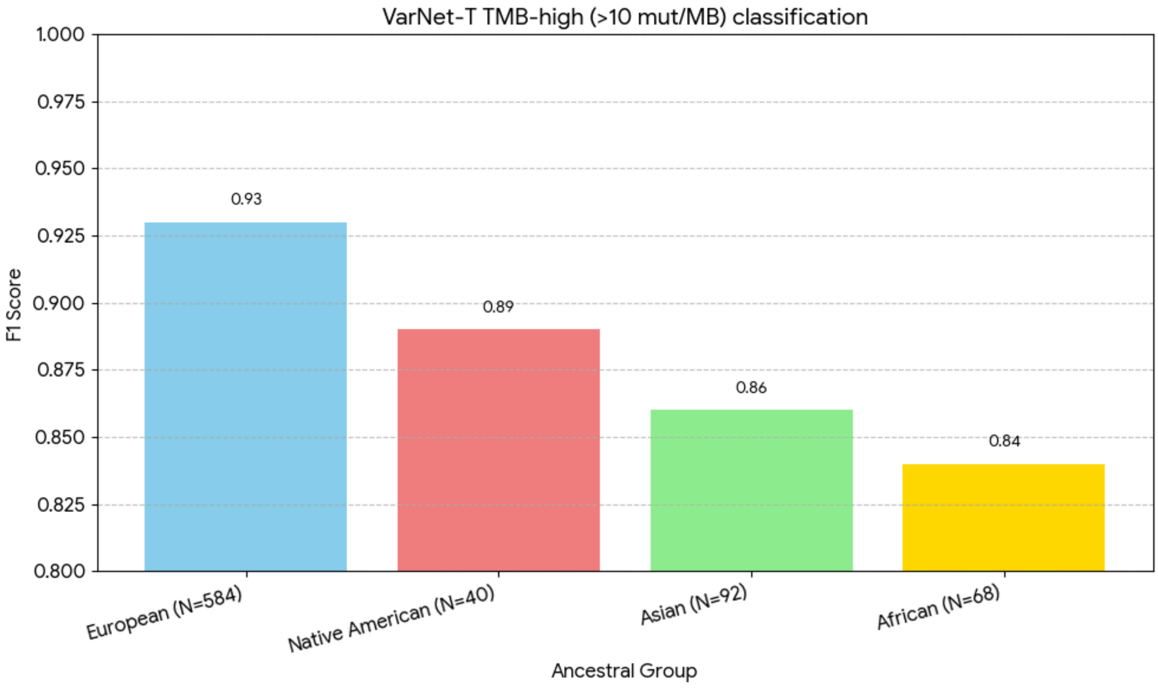
Edits to manuscript	We have added this result to the revised manuscript as Fig. 7e.

Reviewer 1, Major Comment 6

Reviewer Comment	*6. When subsampling SEQC2 to create in silico tumours with varying TMB, were variants subsampled according to VAF?*
Author Response	Thanks for this question. When subsampling the SEQC2 dataset to create in silico tumors with varying TMB, variants were not selected based on their VAF. Instead, variants were randomly selected across the entire range of VAFs to ensure that the VAF distribution remained consistent regardless of the resulting mutation burden. This approach was chosen as we wanted to study the effect of mutation burden on the accuracy of variant calling, rather than the influence of VAF itself.

Reviewer 1, Major Comment 7

Reviewer Comment	*7. Given TCGA was used to train VarNet-T. Can it be reliably used to evaluate tumour-only TMB measures in TCGA?*
Author Response	VarNet-T's performance can be reliably evaluated on TCGA tumor-only TMB measures because we ensured there was no overlap between the training and evaluation sets. VarNet-T was trained on a large and diverse dataset of whole-genome sequencing (WGS) samples (see Fig. 1), a dataset which included only a small number of WGS samples from TCGA, specifically TCGA-LIHC liver samples. These specific TCGA samples used for training were kept entirely separate from the cohort of 1,000 WXS samples that were used

	to perform the TMB evaluation, ensuring that the WXS evaluation set represented unseen data for assessing VarNet-T's performance in that context.

Reviewer 1, Major Comment 8

Reviewer Comment	*8. The authors suggest that matched tumour and normal sequencing allows models to learn shared patterns in sequencing artifacts. They report over 1 million sequencing artifacts in identified in both tumour and normal sequencing from an individual with CLL. Could the authors comment on other characteristics of these putative sequencing artifacts? Are they occurring in repeat regions? At regions of low coverage? Could post-hoc filters aid in removing these variants?*
Author Response	We appreciate the reviewer's insightful question regarding the characteristics of the ~1 million variants identified in both tumor and normal sequencing of the CLL/MBL samples. We performed a post-hoc analysis on these shared sites with respect to common challenging regions:<br><br>● **Repeat regions:** While we observed that a portion of the shared, putatively artifactual sites did fall within repeat regions (as defined by RepeatMasker: https://genome.ucsc.edu/cgi-bin/hgTrackUi?g=rmsk), we found that applying a strict filter on these regions is highly detrimental to sensitivity. Specifically, in the CLL sample, 664 out of 1,319 true somatic SNVs were located within these repeat regions. This demonstrates a significant overlap where over 50% of ground truth mutations are situated in these regions for this CLL sample. This critical overlap means that filtering based purely on repeat region annotations would result in an unacceptable level of false negatives. This finding highlights the necessity of a machine learning model, like VarNet-T, to accurately distinguish true somatic mutations from systemic sequencing noise in these challenging genomic areas.<br>● **Low coverage:** We found that **<15%** of these shared sites had a mean coverage below 30x. This indicates that these artifacts are not simply confined to regions of critically low sequencing depth.<br><br>While established public blacklists might filter a small subset of the ≈1 million shared variants, simple post-hoc metrics like coverage or repeat region annotation may be ineffective or negatively affect accuracy. Hence, these shared sites may represent complex, systemic sequencing noise that is not reducible to simple genomic filters. This presents a challenge for all tumor-only variant callers.

Reviewer 1, Minor Comment 1

Reviewer Comment	*1. Supplementary figures references are out of order and can be confusing to follow at times.*
Author Response	Thanks for bringing this to our attention. We have re-ordered the supplementary figures to be in order of reference.
Edits to manuscript	Updated Supplementary figure numbers and references.

Reviewer 1, Minor Comment 2

Reviewer Comment	*2. In Methods, under Training Data, VarNet is cited as [1] which does not refer to the VarNet citation*
Author Response	Thanks for bringing this to our attention. We have fixed the citation.

Reviewer 1, Minor Comment 3

Reviewer Comment	*3. In the Methods, the authors cite the VarNet manuscript for more details on the data that was used to train VarNet-T. The training data is an important consideration when assessing the model, thus, it is requested that the author restate the training data directly in the Methods of this manuscript.*
Author Response	Thanks for the suggestion, we have included a new training data description.
Edits to manuscript	We have added a description of the training data to the Methods section.

Reviewer 1, Minor Comment 4

Reviewer Comment	*(Remarks on code availability)*<br>*Tried to run docker as outlined in README file, however, the first filter step (filter.py) failed with the error: "filter.py: error: the following arguments are required: --normal_bam". This is likely a small bug with how the command line arguments were set and would just require an update.*
Author Response	Apologies for the error as the VarNet-T code was not yet released to GitHub. You may run VarNet-T using the below commands: (https://github.com/skandlab/VarNet/tree/main?tab=readme-ov-file#new-tumor-only-mode).<br><br># download the latest docker image<br>docker pull kiranchari/varnet:latest<br><br># run VarNet-T filtering:<br><br>docker run -it --rm -v /host:/mount -w /VarNet kiranchari/varnet:latest /bin/bash -c "git pull; python filter.py \\<br>--sample_name test \\<br>--tumor_bam /mount/tumor.bam \\<br>--processes 8 \\<br>--output_dir /mount/output_dir \\<br>--reference /mount/hg38.fa"<br><br># run VarNet-T prediction:<br><br>docker run -it --rm -v /host:/mount -w /VarNet kiranchari/varnet:latest /bin/bash -c "git pull; python predict.py \\<br>--sample_name test \\<br>--tumor_bam /mount/tumor.bam \\

	--processes 8 \ --output_dir /mount/output_dir \ --reference /mount/hg38.fa" <br>The vcf will be generated at /mount/output_dir/test/test.vcf.gz

Reviewer 2 Comments

Reviewer 2, General Comments

Reviewer Comment	*The difference between VarNet-T is quite substantial, yet there isn't a clear explaination as to what specific innovations are responsible for this difference. Deepsomatic, for example, is a conceptually similar application.*
Author Response	We acknowledge the conceptual similarity between VarNet-T and DeepSomatic, but the substantial performance advantage of VarNet-T is fundamentally driven by its key innovation in model training and training data generation. VarNet-T's superior results stem from the creation of a massive training set using real patient-derived tumor samples via weak supervision, allowing it to capture the biological variation and complex tumor heterogeneity - including the presence of normal contamination and subclonality leading to lower VAFs - that are characteristic of real world biopsies. In contrast, DeepSomatic was trained exclusively on cancer cell lines with approximately 40k SNVs, and ClairS-TO used cell-line derived and synthetic mutations, both lacking the biological realism of VarNet-T's training data, which comprises millions of mutations derived from real tumor samples. This dramatic increase in volume and, more importantly, the clinical relevance of the training data is an important reason for VarNet-T's performance and its ability to generalize effectively to patient data.
Edit to manuscript	We have emphasised the key innovation of VarNet-T's training on a large number of real tumor-derived mutations using weak supervision as compared to other methods such as DeepSomatic and ClairS-TO (see Introduction and Results).

Reviewer 2, Major Comment 1

Reviewer Comment	*Was the subtraction of germline variants (via gnomAD) done with the same variants across all tumor-only callers? The methods only mention this being done for VarNet-T.*
Author Response	VarNet-T, Mutect2, ClairS-TO and DeepSom all utilize the germline variants from gnomAD for subtraction/filtering. DeepSomatic deviates from this approach; rather than simple filtering, it integrates the population allele frequency (AF) of variants into its model by encoding it as a dedicated channel. This difference in methodology helps DeepSomatic achieve higher sensitivity, although typically at the cost of reduced precision.

Reviewer 2, Major Comment 2

Reviewer Comment	*If the germline pre-filtering procedure (Supplemental Table 3) is applied to VCFs from other tumor-only methods do their metrics improve substantially?*

Author Response	VarNet-T, Mutect2, ClairS-TO and DeepSom already utilize gnomAD-based filtering strategy. DeepSomatic employs an integrated design where the population allele frequency (1000 Genomes AF) is encoded directly as a model channel - not applied as a simple post-filter. This design is a core innovation and is already optimized for sensitivity. However, as suggested by the reviewer, when we applied our germline filtering procedure to DeepSomatic's VCFs for the public benchmark reference datasets, we observed an improvement in its metrics although VarNet-T still performs better (see table below). We excluded SEQC2 as DeepSomatic was trained on SEQC2 sample.

Method	COLO829 (AUPRC)	AML (AUPRC)	ICGC CLL (AUPRC)	ICGC MBL (AUPRC)
VarNet-T	0.656	0.112	0.032	0.062
DeepSomatic	0.118	0.004	0.003	0.005
DeepSomatic + VarNet-T's germline filtering	0.474	0.087	0.030	0.062

Reviewer 2, Major Comment 3

Reviewer Comment	*217-228: The improvement represented by VarNet-T in estimating TMB is substantial, requiring an explaination for the improvement over other methods. What mutation tumour/normal mutation caller was used for TMB estimation in figure 4 and supplemental figs 8-17? Are these results sensitive to the choice of tumor-normal variant caller?*
Author Response	Thanks for this question. In the TMB analysis, we used VarNet as the tumor-normal mutation caller as independent benchmarking has shown that VarNet is an accurate variant caller in the tumor-normal setting [Krishnamachari et al., Nature Communications 2022]. We also tested TMB-High (>=10 mut/MB) status classification using Strelka2 as the baseline tumor-normal caller [Kim, S et al. Nature Methods 2018]. This analysis yielded similar results, with VarNet-T achieving the best performance compared to other tumor-only methods (figure below).

	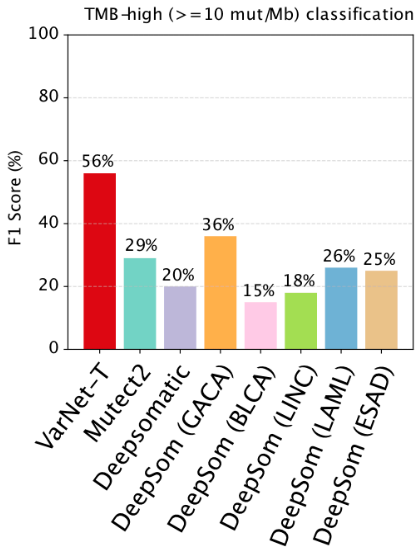

Reviewer 2, Major Comment 4

Reviewer Comment	*For other ML-based tools (e.g. deepvariant) is there an indication of how many examples went into training the model vs the number that went into VarNet-T?*
Author Response	We appreciate the question. DeepSomatic was trained on the SEQC2 sample, which provides a high-confidence ground-truth set of approximately 40,000 single nucleotide variants (SNVs). ClairS-TO, another recent deep learning method, employed a more expansive approach, utilizing millions of *in silico* generated synthetic mutations augmented by tens of thousands of real mutations derived from cancer cell lines. VarNet-T, however, introduces a key innovation: its training utilized millions of real somatic mutations derived directly from patient tumor samples. This biologically realistic training set allows VarNet-T to effectively capture the complex biological variation, tumor heterogeneity, and sub-clonality observed in real-world biopsies. This contrast underscores a crucial point: superior performance is not solely determined by the quantity of training data, but by its biological realism.

Reviewer 2, Major Comment 5

Reviewer Comment	*Even when carried out with the best of intentions, benchmarking by the authors will naturally be biased towards the tool the authors are presenting given greater familiarity around parameterisation. Would it be possible for a comparison of results previously obtained by authors of a leading competing method?*
Author Response	We understand the concern regarding parameterization bias; however, none of the benchmarked methods (VarNet-T, Mutect2, DeepSom, DeepSomatic and ClairS-TO) require manual tuning of parameters and are run using their published default settings, mitigating the risk of favorable adjustment. Moreover, the published benchmarks of DeepSomatic [Park, J. et al. Nature Biotechnology] and DeepSom [Vilov, S. & Heinig, M.,

	Bioinformatics] do not include whole genome evaluations using the same public reference datasets included in our study. For instance, DeepSomatic trained its model on the SEQC2 dataset (excluding chromosome 1) and evaluated it only on the held-out chromosome 1, which prevents a direct, apples-to-apples comparison with our analysis. Mutect2 [Benjamin, D. et al., bioarxiv] manuscript does not include tumor-only benchmarks. While ClairS-TO [Chen, L. et al., Nature Communications] includes a short-read benchmark on the COLO829 sample, the authors applied custom filters to the truth set, making a direct comparison to their published results unfeasible. Our study overcomes these limitations by using whole-genome evaluations and unmodified public truth sets for a more robust and direct comparison.

Reviewer 2, Remarks on code availability

Reviewer Comment	*Software should be released on github with more extensive instructions on its use. This needs to be made more accessible e.g. when posted to github, instructions need to include more examples around different use cases and detailed installation instructions in addition to test data to verify a users's installation. The code itself includes hard-coded paths local to the authors and other artefacts that should be cleaned up prior to release.*
Author Response	Thanks for the suggestion. We have updated the GitHub repository. There is no installation required as the provided docker image includes all required libraries and code pre-installed. VarNet-T can be run by simply omitting the normal sample argument in VarNet (https://github.com/skandlab/VarNet/blob/main/README.md#new-tumor-only-mode). We have included example commands in the README. Please use the latest docker image/commands for running VarNet-T.<br><br># download the latest docker image<br>docker pull kiranchari/varnet:latest<br><br># run VarNet-T filtering:<br><br>docker run -it --rm -v /host:/mount -w /VarNet kiranchari/varnet:latest /bin/bash -c "git pull; python filter.py \\<br>--sample_name test \\<br>--tumor_bam /mount/tumor.bam \\<br>--processes 8 \\<br>--output_dir /mount/output_dir \\<br>--reference /mount/hg38.fa"<br><br># run VarNet-T prediction:<br><br>docker run -it --rm -v /host:/mount -w /VarNet kiranchari/varnet:latest /bin/bash -c "git pull; python predict.py \\<br>--sample_name test \\<br>--tumor_bam /mount/tumor.bam \\<br>--processes 8 \\<br>--output_dir /mount/output_dir \\

	--reference /mount/hg38.fa"<br><br>The vcf will be generated at /mount/output_dir/test/test.vcf.gz

We thank the reviewers for their positive feedback and constructive comments to improve the manuscript. We have made point-by-point responses to all the reviewers' comments and descriptions of the corresponding changes to the manuscript below. All modified text and figures in the manuscript and supplementary sections are in blue for ease of review.

Reviewer 1 Comments

Reviewer 1, General Comments

Reviewer Comment	*I thank the reviewers for their thoughtful responses to my comments. My comments have largely been addressed. I have only two minor remaining items:*
Author Response	We thank the reviewer for the positive feedback and suggestions to improve the manuscript. Below we provide a detailed point-by-point response for all the comments.

Reviewer 1, Minor Comment 1

Reviewer Comment	*1. In response to a few comments, it appears that the new data was only included in the response to the reviewer not in the manuscript itself. I believe these analyses/data (even if not beneficial to model performance) would still be of interest to readers and support the parameterization choices made. Could the authors add the following to the manuscript in supplementary:*<br>*a. Could the authors please incorporate the data showing the sensitivity by mutational process into the supplementary of the manuscript (Comment 3)?*<br>*b. Similarly, could the authors add the model performance when trained with different proportions of somatic and non-somatic variants to the manuscript (Comment 2)?*<br>*c. Finally, could the authors include a statement about the limitations (i.e. the reduction in sensitivity) of post-hoc genomic filters in the manuscript (Comment 8)?*
Author Response	Thank you for the suggestion to improve the manuscript. We have included these sections as supplementary notes 1,3 and 4 and referenced them in relevant parts of the manuscript.
Edits to manuscript	We also examined mutational context bias in variant calling. While the VarNet-T base deep learning model maintained consistent sensitivity across all single base substitution (SBS) contexts, applying germline (gnomAD/dbSNP) and Panel-of-Normals (PoN) filters notably reduced sensitivity for C>T transitions (Supplementary Note 1). This bias stems from the high prevalence of C>T variants in both germline databases and sequencing artifacts. This highlights the trade-off in using public databases for germline exclusion.<br><br>...<br><br>The difficulty in distinguishing artifacts in the absence of a matched normal sample is further illustrated in Supplementary Note 4, where we show that training VarNet-T on different distributions of somatic versus non-somatic variants—even upweighting

non-somatic variants to better represent a higher artifact-to-somatic ratio—resulted in poorer performance on low tumor mutation burden (TMB) samples.

…

Supplementary Note 1

Impact of Mutational Context on VarNet-T Sensitivity

We calculated VarNet-T's sensitivity for different mutational processes (SBS, Single Base Substitution) using the SEQC2 benchmark dataset. The VarNet-T base model did not display any significant bias towards any one mutational context as it achieved balanced accuracy across all contexts (Supplementary Fig. 17a). However, after applying variant and artifact filtering using gnomAD/dbSNP/Panel-of-Normals, there were some mutational contexts (specifically C>T changes) where sensitivity was lower (Supplementary Fig. 17b).

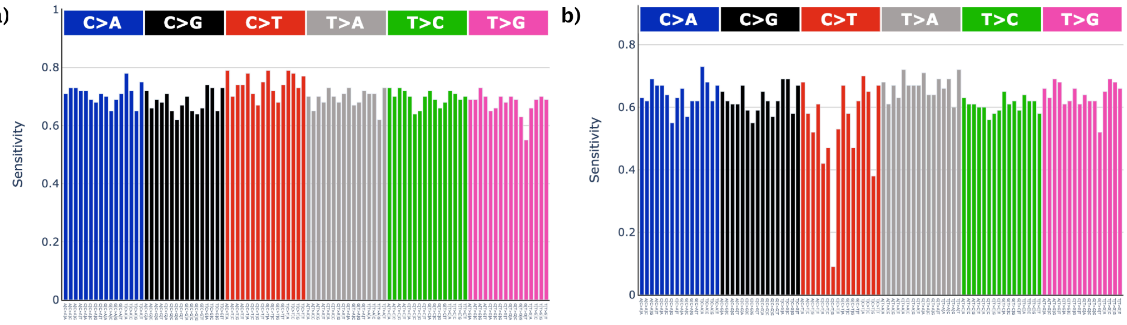

Supplementary Fig. 17: VarNet-T's SNV calling sensitivity across mutational contexts on the SEQC2 sample. a without applying gnomAD-dbSNP-PoN filtering **b** after applying gnomAD-dbSNP-PoN filtering. Source data are provided as a Source Data file.

This is attributable to the fact that this is a common germline as well as artifact signature. Indeed, we measured and plotted the frequency of each mutation context in gnomAD and the 1000 genomes panel of normals and found that C>T variants have the highest proportion in these germline datasets (Supplementary Fig. 18).

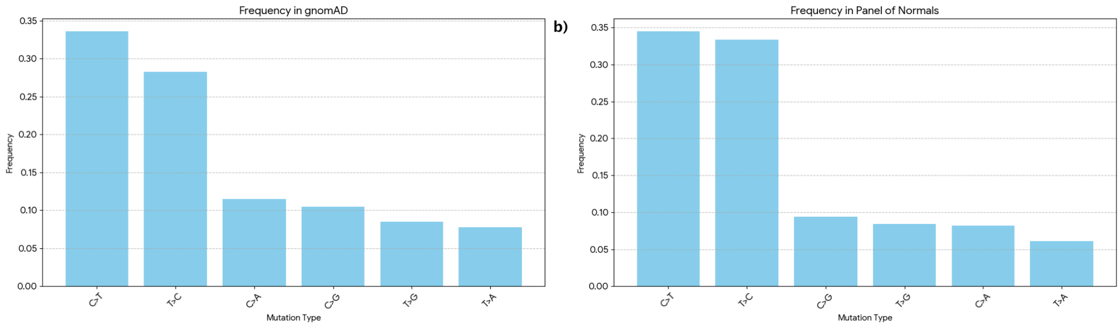

Supplementary Fig. 18: Frequency of SNV base changes observed in **a** gnomAD and **b** 1000 genomes panel of normals. Source data are provided as a Source Data file.

This observed drop in C>T sensitivity is not unique to VarNet-T, as similar performance biases against C>T variants are also seen in other tumor-only methods employing gnomAD

filtering, such as Mutect2 and DeepSom (Supplementary Fig. 19).

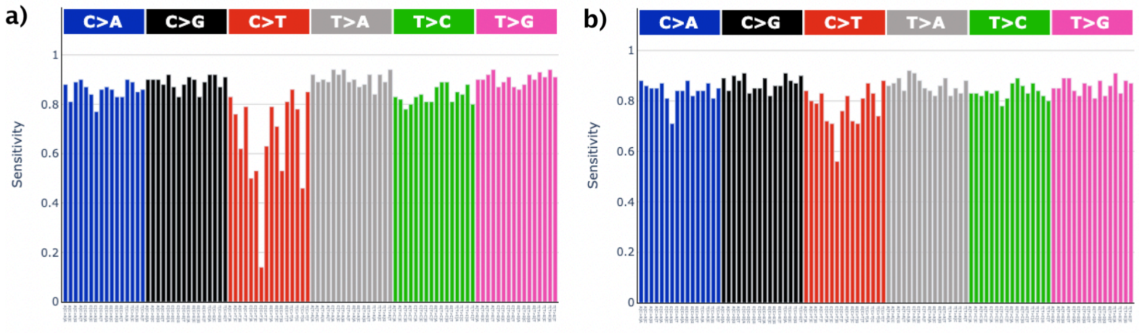

<u>Supplementary Fig. 19:</u> SNV calling sensitivity across mutational contexts on the SEQC2 sample. a DeepSom **b** Mutect2. Source data are provided as a Source Data file.

Supplementary Note 3

<u>Shared sequencing artifacts between matched tumor and normal samples</u>

We hypothesized that a significant proportion of sequencing artifacts observed in tumor samples are also present in the matched normal samples. This overlap allows tumor-normal variant callers to identify low-frequency sequencing artifacts occurring in both tumor and normal samples. To test this hypothesis, we extracted all SNV variant candidates (VAF > 3.5%) from the matched normal and tumor samples of CLL and MBL individually. A germline variant caller was then applied to each matched normal sample, and germline variants were removed from the variant candidates in both the normal and tumor samples for the corresponding pairs. Additionally, ground-truth somatic SNVs were excluded from the variant lists.

The remaining variants in each normal and tumor sample are presumed to predominantly represent sequencing artifacts, as germline and somatic events had been filtered out. In the CLL sample, we identified 1,525,250 and 1,352,545 putative sequencing artifact variants in the matched normal and tumor samples, respectively. Of these, 939,379 variants were shared between the matched normal and tumor samples, indicating that 70% of the sequencing artifact variants observed in the tumor sample were also present in the matched normal sample. As expected, the VAF distribution of these shared sequencing artifacts was lower than that of shared germline mutations (Supplementary Fig. 21). These findings support our hypothesis that the matched normal sample can aid in filtering shared sequencing artifacts in the tumor sample.

A similar pattern was observed in the MBL sample, where 64% of sequencing artifacts detected in the tumor sample were also found in the matched normal sample.

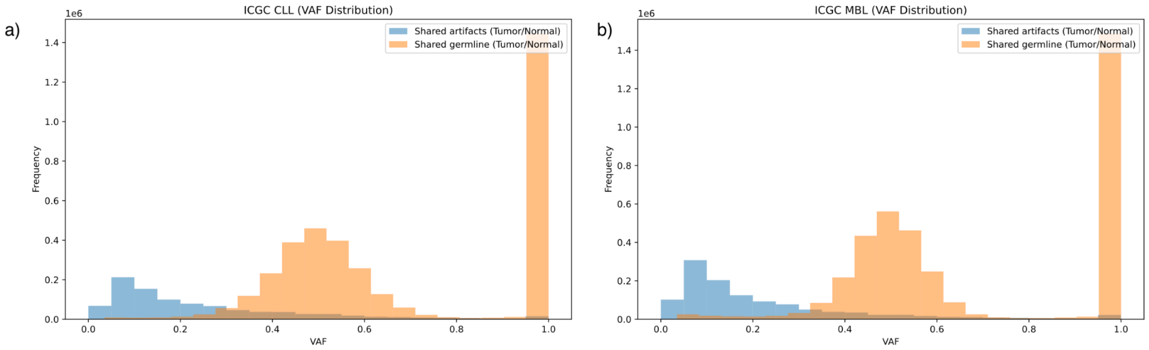

<u>Supplementary Fig. 21:</u> VAF distribution of shared sequencing artifacts and germline mutations between matched normal and tumor samples.

We further performed a post-hoc analysis on these shared artifact sites with respect to common challenging regions. While we observed that a portion of the shared, putatively artifactual sites did fall within repeat regions (as defined by RepeatMasker: https://genome.ucsc.edu/cgi-bin/hgTrackUi?g=rmsk), we found that applying a strict filter on these regions is highly detrimental to sensitivity. Specifically, in the CLL sample, 664 out of 1,319 true somatic SNVs were also located within these repeat regions. This demonstrates a significant overlap where over 50% of ground truth mutations are situated in these regions for this CLL sample. This critical overlap means that filtering based purely on repeat region annotations would result in an unacceptable level of false negatives. This finding highlights the necessity of a machine learning model, like VarNet-T, to accurately distinguish true somatic mutations from systemic sequencing noise in these challenging genomic areas. We also found that only <15% of these shared sites had a mean coverage below 30x. This indicates that these artifacts are not simply confined to regions of critically low sequencing depth. While established public blacklists might filter a small subset of the ~1 million shared artifactual variants, simple post-hoc metrics like coverage or repeat region annotation may be ineffective or negatively affect accuracy. Hence, these shared sites may represent complex, systemic sequencing noise that is not reducible to simple genomic filters. This presents a challenge for all tumor-only variant callers.

Supplementary Note 4

<u>Impact of Training Variant Distribution on VarNet-T Performance</u>

We trained two models to reflect a different distribution of somatic vs non-somatic variants by upweighting non-somatic variants during training. We trained one model to reflect a ~33% somatic vs ~66% non-somatic variants and another model with ~20% somatic vs ~80% non-somatic variants. Both these models performed worse than our baseline VarNet-T model (~50% somatic vs ~50% non-somatic variants) on the low TMB ICGC CLL/MBL benchmark samples (Supplementary Table 6). These results suggest that the difficulty is primarily in distinguishing artifacts in the absence of a matched normal sample.

Model	MBL (AUPRC)	CLL (AUPRC)
VarNet-T (50% somatic vs 50% non-somatic)	0.032	0.062
VarNet-T (~33% somatic vs ~66%	0.029	0.060

	non-somatic)		
	VarNet-T (20% somatic vs 80% non-somatic)	0.027	0.056
	**Supplementary Table 6:** Performance of VarNet-T trained on different proportions of somatic vs non-somatic variants on low mutation burden tumors. Source data are provided as a Source Data file.		

Reviewer 1, Minor Comment 2

Reviewer Comment	*2. Regarding the use of TCGA in the training data, I appreciate the authors only considered a subset of tumours with WGS in training leaving 1,000 WXS samples for testing. However, some donors in TCGA have both WXS and WGS profiling. Could the authors please confirm that no donors overlap their training and testing cohorts and include a statement in the manuscript?*
Author Response	Thanks for the suggestion, we have confirmed that there is no overlap between training and test cohorts and included a list of the TCGA patient IDs used for training and testing in the supplementary file (TCGA_samples.xlsx).
Edits to manuscript	We have included a statement under Methods > TCGA sample selection for TMB estimation.<br><br>Samples used in the training cohort were excluded from this analysis. A comprehensive list of the TCGA sample IDs utilized for training and testing can be found in the supplementary file (TCGA_samples.xlsx).
